# Energy efficient drying technologies for sweet potatoes: Operating and drying mechanism, quality-related attributes

**DOI:** 10.3389/fnut.2022.1040314

**Published:** 2022-10-20

**Authors:** Muhammad Tayyab Rashid, Kunlun Liu, Mushtaque Ahmed Jatoi, Bushra Safdar, Dingyang Lv, Qingyun Li

**Affiliations:** ^1^College of Food Science and Engineering, Henan University of Technology, Zhengzhou, China; ^2^Department of Botany, Shah Abdul Latif University, Khairpur, Pakistan; ^3^Beijing Advanced Innovation Center for Food Nutrition and Human Health, Beijing Technology and Business University (BTBU), Beijing, China

**Keywords:** sweet potatoes, drying methods, kinetics, mathematical modeling, energy considerations

## Abstract

Sweet potatoes (SPs) are a versatile tuberous crop used as subsistence and cash crop in raw and processed forms. The major issue with SPs is post-harvest losses, which result in noticeable quality decline because of inappropriate handling, storage, delayed transit, and sales, as well as microbiological and enzymatic activity. Drying is an excellent strategy for managing short postharvest storage life, preserving nutrients, and maximizing long-term benefits. However, several parameters must be considered before drying SPs, such as relative humidity, temperature, drying duration, size, and shape. The current review looks at the factors influencing SPs' moisture loss, drying kinetics, diverse drying methods, pretreatments, operating conditions, and their efficacy in improving the drying process, functional, and nutritional qualities. An optimal drying process is required to preserve SPs to obtain concentrated nutrients and improve energy efficiency to be ecofriendly. Drying sweet potatoes using traditional methods such as sun or open-air drying was found to be a slow process that could result in a lower quality. Various advanced drying techniques, like vacuum, infrared, freeze drying, and pretreatments such as ultrasound and osmotic dehydration, have been developed and are successfully used globally. The best-fit thin-layer models (Hii, Page, two-term, logarithmic) utilized for drying SPs and appropriate modeling methods for optimizing drying procedures are also discussed.

## Introduction

**Figure 1:**
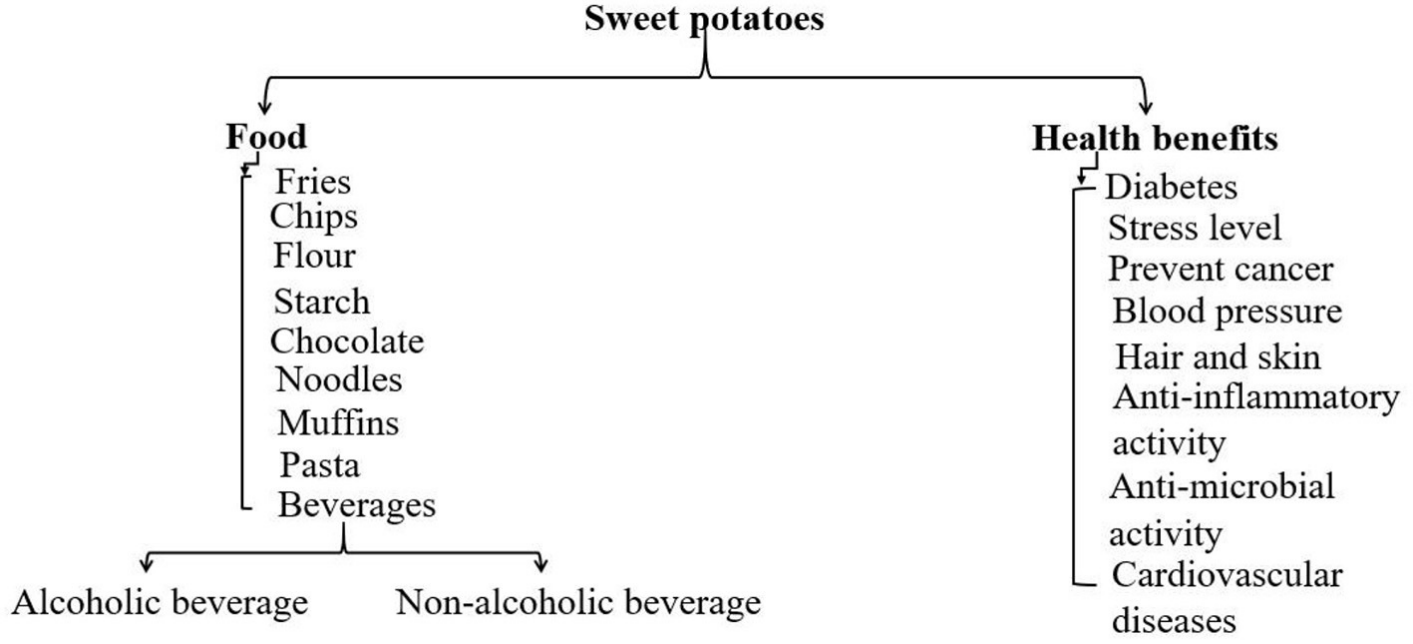
Classification of and health benefits of dried sweet potato products.

The sweet potato is a tuberous vegetable that is a member of the family Convolvulaceae, cultivated in numerous Asian countries due to its ease of growth and high productivity. The leading producers include China, Indonesia, Nigeria, Uganda, and Vietnam (1). Sweet potato tubers contain macronutrients like starch, fiber, and protein, as well as a variety of micronutrients including minerals (manganese, copper, potassium, and iron), vitamins (primarily B complex, C, and E), and provitamin A (as carotenoids), anthocyanins (purple sweet potatoes), flavonoids, and coumarins (2). When compared to other root and tuber crops, the sweet potato contains more carbohydrates and proteins, as well as certain vitamins and minerals, and it contains more provitamin A, vitamin C, and minerals than wheat or rice (3). Due to its high concentrations of bioactive secondary metabolites, sweet potatoes are gaining popularity among the food industry, consumers, and scientists, not only as a healthy product but also as an ingredient for functional foods. The consumption methods and health-associated benefits of sweet potatoes are listed in Figure 1. It is considered a traditional food crop SPs are classified as perishable if their moisture level exceeds 70% (4). As the higher moisture level makes them particularly predisposed to microbial decay, even when stored at room temperature (5). The postharvest behavior of sweet potatoes is sensitive to storage under ambient and cold temperatures due to sprouting and chilling injuries, respectively. An effective postharvest preservation method such as drying is recommended to expand the shelf life of SPs besides processing them into various by-products. Drying can be performed as a unit operation in agricultural crop processing (6).

Drying is a mass transfer phenomenon, involving the evaporation of water from food. It entails applying heat to a material, which causes internal moisture to be transferred to the surface of the material, which is then removed into the atmosphere (7, 8). It is the conventional and most common way of preserving food, increasing shelf-life and product quality, minimizing the weight and bulk, lowering handling and packaging efforts, as well as freight costs. The airflow promotes heat application by generating and eliminating humidity. The drying process most importantly moisture content, can be easy to be removed when the relative humidity is lower. The dried products retain nutrients, color, and fragrance if the drying process is appropriately controlled. The efficient drying of fruits and vegetables allows consumers to enjoy these delicacies even during the off-season.

Food drying includes traditional (sun or wind-drying), and non-traditional methods (infrared, vacuum drying, microwave, and freeze drying). Modern drying techniques allow uniform drying in a shorter period and extended storage life of up to a year or more. Recent advancements have produced drying processes that focus on improving existing methods to deliver high-quality goods using less energy, such as freeze-drying, vacuum, microwave, and microwave-combined freeze-drying (9). Refractance window drying is a popular method for converting liquid food and other biomaterials into high-value powders, flakes, or sheets. This drying technique is simple and affordable for freeze-drying, but it requires large installations to be cost-effective (10). Finally, microwave drying can reduce drying time and thus enhance the quality of the final product (11).

There are many studies on sweet potato drying, however, they lack collective information that would help researchers who want to carry out more research. Similarly, different factors must be considered, such as drying methods, pretreatments, kinetics, nutrient degradation, energy activation, and mathematical modeling. The myriad factors make it difficult for researchers and concerned food industries to understand the pros and cons of the technologies to adapt an appropriate method for drying SPs. This study aims to provide a comprehensive review of the influence of numerous drying techniques on the water loss ratio of SPs and simplify the drying kinetics and other necessary operating conditions to achieve quality and nutrient preservation in the final product. The key objectives are to explore the impact of operating conditions in drying SPs, appropriate drying methods, osmotic and chemical pretreatments, effects of drying processes, nutritional qualities, models that are most suited for drying, energy consumption, and further improvement with the recent trends in SPs drying techniques.

## Sweet potato drying approach

The drying process begins with removing unbound surface moisture and progresses through the removal of bonded humidity inside the food material until a predefined limit is achieved. Mass and heat are transferred at the same time in the drying. Agricultural commodities require a favorable drying environment, high temperature, and a high capacity of air to absorb water or low relative humidity during the operation.

**Figure 2:**
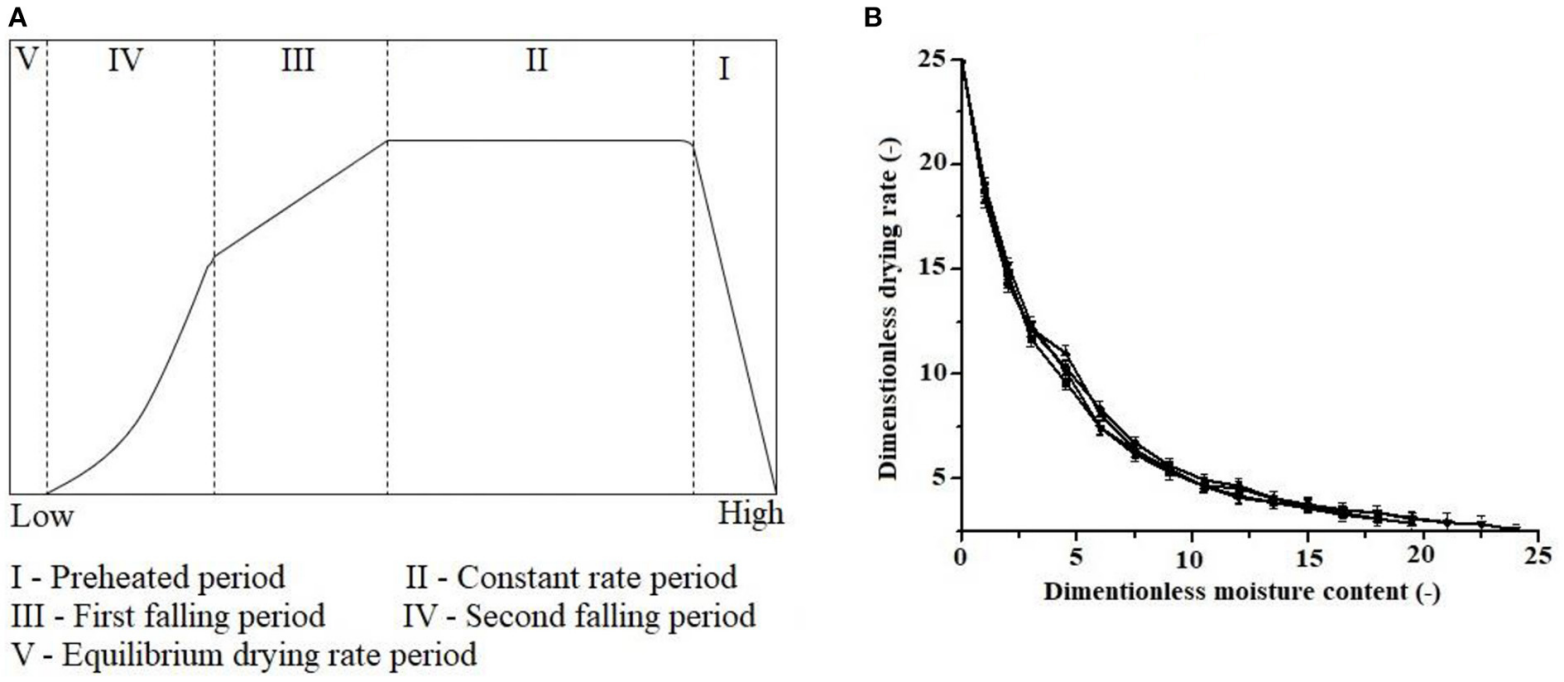
**(A)** Typical stages of food product drying, **(B)** drying curve of sweet potatoes.

The drying process of sweet potatoes product comprises four stages after a preheating period, as depicted in Figure 2A. Stage I, with a constant drying rate, refers to the frame of energy absorption for the moisture release, followed by a stage of evaporation at a decreasing rate. Both are key factors influencing the complete drying mechanism (12). During stage II, evaporation occurs from the food material's external surface to remove physically attached moisture (free water) at a fixed drying rate. This phase did not observe during sweet potato drying due to an insignificant amount of unbound moisture in SPs (Figure 2B). For SPs, stages III and IV show a declining drying rate until the anticipated moisture content is attained. When the transportation of moisture from the inner of the material to the outer surface (low concentration gradient) slows down (as compared to the evaporation at previous stages at a constant rate) and results in a decline in drying rate. When the equilibrium moisture content is achieved, no further moisture exchange occurs between the material and adjacent air, which is the end of the drying process. Dehumidified air at high temperatures can be used to continue drying SPs below the equilibrium moisture content.

## The role of operational conditions in drying sweet potatoes

Improper drying often harms the quality and nutritional profile of a food item. Therefore, optimal drying conditions are necessary to produce prime-quality dried SPs with their optimum nutritive and functional potential. To optimize the drying method for SPs, certain factors must be considered, including slice thickness, drying time, and climate conditions. Furthermore, to get the anticipated moisture level for the food materials, the relative humidity under isothermal conditions can be appropriately controlled by drying techniques. Some industrial dryers suitable for drying SPs are listed in Tables 1–**4**, along with their effects on the drying process' effectiveness.

**Table 1:** Hot air-drying (HAD) studies were conducted to dry sweet potatoes.

**Drying conditions/Temperature (°C)/Geometry**	**Size (mm)**	**Response**	**Main conclusion**	**References**
HAD; *T* = 50–90°C; *V* = 1.0 m/s; *RH* = 50%; *G* = Slices	3–8	Thin layer models, D*_*eff*_, E_*a*_*	Hii model showed results for D*_*eff*_* (3.66 10^−10^ to 2.11 10^−9^ m^2^/s) and *E*_a_ (13.48–16.40 KJ/mol)	(13)
CD; *T* = 50–90°C; *V* = 1.5 to 5.5 m/s; *RH* = 23 and 50%; *G* = Cubes	5–12	Thin layer models, *D_*eff*_, E_*a*_*	*E_*a*_* was 11.38 KJ/mol; D*_*eff*_* increased with temperature. Page Model performed better	(14)
TD and FBD at 70 and 80°C; *G* = Cylinders	–	Drying, blanching, shrinkage, color	Fluidized bed dryer temperature didn't affect shrinkage, quality and appearance were better, and blanching improved color	(15)
CFD = 42°C, SD = 27–50°C; *G* = Slices	–	Provitamin A carotenoid analysis, moisture/water activity	Solar and sun drying retained Provitamins A. Vitamin A was rich in orange sweet potato flour	(16)
Tunnel drying; *T* = 60–80°C; *V* = 0.42 to 1.12 m/s; *RH* = 10–15%; *G* = Slices	–	Mathematical modeling, *E_*a*_*	The logarithmic model best fits drying data. *E_*a*_* = 23.29 KJ/mol	(17)
OD; *T* = 50–80°C; *G* = Slices	5, 10, 15	Mathematical modeling, *E_*a*_*	Page model and modified page model best described drying. *E_*a*_* = 11.10–30.40 KJ/mol	(18)
HAD; *T* = 40°C, *V* = 1 m/s; *G* = Slices	5	Hyperspectral imaging, modeling	Hyperspectral imaging was a fast way to predict moisture content, and the RC-MLR model was best	(19)
HAD; *T* = 50–90°C; *G* = Slices.	5, 8, 12	Neutral network, MC, drying kinetics	ANN models can be used to estimate drying online	(20)
DD; *T* = 120, 130 and 140°C; *G* = Slices	–	Phytochemicals and antioxidants	Higher TPC and antioxidant activities at 140°C and drum-dried flour have higher antioxidants	(21)
CD; *T* = 50, 60, and 70°C; *G* = Slices	30 × 48 × 5	Drying kinetics, rehydration ratio	The logarithmic model best fit with *E_*a*_* at 23.2 KJ/mol	(22)
TD; *T* = 50–80°C; *V* = 2.5 m/s; *RH* = 10%; *G* = Slices	–	Drying kinetics, blanching	Page model fits best, and blanched samples dried faster than unblanched	(23)

HAD, hot air dryer/drying; CD, cabinet dryer; TD, tray dryer/drying; OD, oven dryer/drying; DD, drum dryer/drying; ANN, neutral network approaches; US, ultrasound; *D*_*eff*_, moisture effective diffusion/moisture diffusivity; E_*a*_, activation energy; SEM, scanning electron microscopy; TPC, total phenolic content.

### Relative humidity

Relative humidity (RH) is the ratio of saturated to humid air's water vapor pressure. It is an important drying parameter that affects the transport of mass and heat as well as the quality and efficiency of drying. It shows the degree of divergence from saturated moist air and the moisture absorption ability of the drying medium moist air. However, the mechanism by which RH influences drying behavior is unclear. As a result, there is no definitive RH control strategy for drying fruits and vegetables. It was found that continuous dehumidification during drying may increase drying efficiency, and the lower the RH level, the faster the drying rate, as observed in rapeseed and spaghetti (59, 60). Only a few studies have considered the effect of RH in sweet potato drying, thus optimizing RH is still necessary in this case. According to Sun et al. (19), the elevated drying temperature may reduce the RH of the drying medium and increase the vapor pressure shortfall, boosting the degree of external mass transfer. Similarly, Sabudin et al. (61) discovered that 40% relative humidity had the highest drying rates and moisture gradient in sweet potato drying.

### Air velocity

The uniform air circulation defines the drying operation duration and the final dried product quality. Generally, hot air maintains a specified temperature range (40–50°C) in the dryer throughout the drying process. The surrounding fresh air absorbs the moisture from the product's outer surface with low RH%, which increases moisture removal efficiency. The airflow over the product's surface ensures rapid moisture removal by evaporation and the maximum drying rate at a constant air temperature and changing ambient RH. Fast air circulation has a high potential to replace the stationary boundary layer of drying air from the surroundings of a humid product. SPs might exhibit a similar phenomenon under higher air velocity. A high drying rate is not usually suggested since it might harm the food matrix, causing cracking or deformation. Laminar airflow might be observed when using a low-speed fan, allowing air passage into the stack, leading to ineffective distribution and lower heat exchange between the air stream and product surface. Walker (62) and Zhu and Jiang (17) noted that the drying operation of SPs is highly influenced by air velocity. In the Wang and Singh model, air velocity and slice thickness are substantial (18).

### Drying time

Drying time and air temperature are inversely related; lowering drying time requires increasing the drying air temperature. The heat transfer increases in direct proportion to the moisture gradient, speeding up the drying. It is attained by the amount of heat provided to the material and moist air is removed from the surrounding area. The drying operation of drying is mainly propelled by the moisture gradient. A higher gradient along the equilibrium might result in faster drying. Otherwise, the absence of a sufficient gradient can cause a reduction in the drying rate and, consequently, may extend the drying period. Significant quality degradation, color deterioration, and structural changes can occur because of prolonging the drying time. Sample thickness, wither degree, temperature, and air velocity are critical parameters for calculating the time needed to produce dried SPs. The continuous moisture removal decreases the moisture content as the drying operation proceeds (13). Using de-humidified drying air and low temperature can enhance the degree of drying and reduce the drying period capture oxidation. During this drying period, the rate of evaporation increases significantly. Retaining the quality and flavor to a high degree is a promising outcome for the obtained product. Furthermore, the product has a longer shelf life and might be used after a long time without degradation.

### Thickness of slices

The size of slices, especially the thickness, is an equally important parameter in the drying operation. A reasonable depth of sweet potato slices is critical to circulate drying air through the voidage from channels, particularly in a fixed bed, conventional, low-capacity tray batch dryer. Thin packable layers are commonly employed to disperse food ingredients. The thick loose layers need for large food items. These materials can induce airflow resistance and may require slightly more time for drying. The transportation of the internal moisture becomes extremely tough in thick slices, owing to decreased mobility in the food matrix, resulting in a lower moisture removal rate. Producing dried SPs with the desired moisture content of <7% requires a very long drying process and often generates uneven products because of insufficient contact between incoming hot air and sweet potato slices (15). Overloading can cause a longer drying time with a need for an expansion in air temperature. The degree of wither and inlet temperature significantly influence the calculation of the thickness and pitch of the spread. Drying time may be reduced, and low-frequency agitation applied to the drier bed can enhance the quality of dried SPs.

### Rehydration

Rehydration is a complicated procedure that aims to restore the raw product's properties. Rehydration capacity is influenced by drying processes and other parameters such as rehydration duration, product composition, and water temperature. A key step in producing dried foods is quick and thorough rehydration. Moreover, drying conditions, pretreatments, and structural properties of dried products can significantly affect the rehydration capacity (63). Water content slowly decreases during the drying process, resulting in irreversible cell damage and displacement, loss of cell integrity, structural collapse, and loss of hydrophilicity. The rehydration index can be determined using Equation (1):


(1) $$\begin{array}{l} R=\left(\frac{W_{d}}{W_{r}}\right)\times 100, \end{array}$$

W_d_ and W_r_ are the dehydrated and rehydrated sample masses (g).

As drying temperature increases, the rehydration index increases as well. The contraction causes the sweet potato's cellular structure to be very porous, enabling more water absorption. SPs demonstrated the least water adsorption by drying at 40°C, while those at 80°C demonstrated the quickest adsorption, perhaps because of decreased shrinkage (63). According to Doymaz (64), SPs have a high moisture content. As a result, water molecules enter SPs with relatively little driving force.

## Classification of drying methods for sweet potatoes

**Figure 3:**
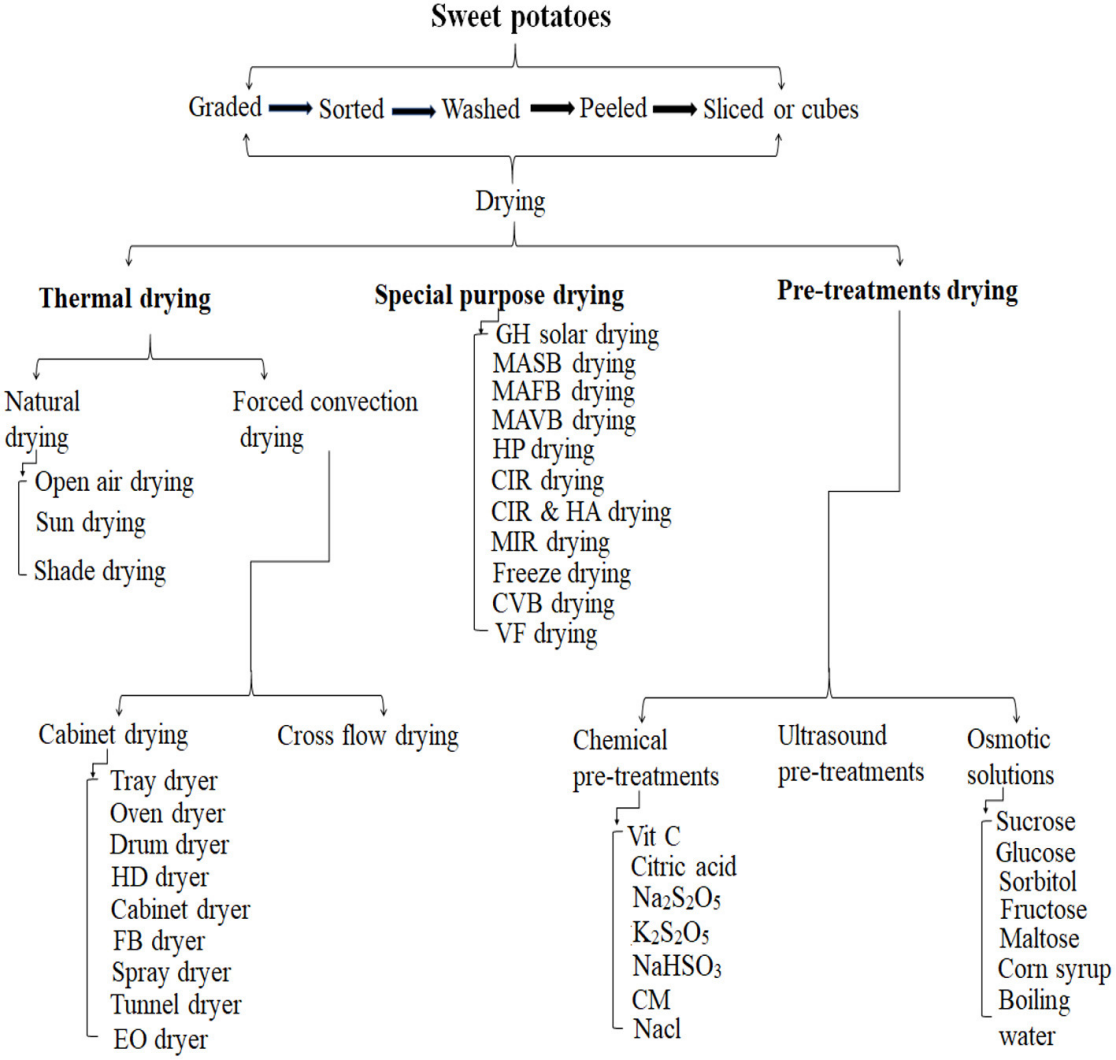
Classification of different drying methods used for sweet potatoes.

SPs can be dried in various ways, as seen in Figure 3. Table 1 contains descriptions of the drying processes.

### Fluidized bed drying

Wet particle drying is accomplished by utilizing FB dryers. Additionally, suspensions, granular materials, pastes, and slurries, can also be fluidized in inert solids bed. FB dryers improve the efficiency of high heat and mass transfer, uniform moisture reduction, good solid mixing, and accessible material transportation (65). They maintain a consistent bed temperature throughout the drying process and extend the constant drying rate period. However, due to high moisture variation, stratified flow and hotspot formation in FB dryers can cause product damage and quality loss. Special additives are required to handle materials and sticky hygroscopic products in an FB dryer. However, a significant drawback of FBs is the chance of a decline in particle size reduction owing to collision and attrition (66). FBD is one of the most effective drying techniques when solely considering thermal efficiency (67). It is the most effective drying technique for granulated materials because its mixing features promote intense mass and heat transference, resulting in a short drying period (36). Hatamipour et al. (15) explored the drying characteristics and kinetics of six sweet potato varieties and concluded that FBD produces excellent quality and nutritional values for all types. Similarly, a study conducted by (36) on SPs showed that the effective diffusion rate (D_*eff*_) in dry beds from 45 to 65°C was 4.92 × 10^−7^-7.26 × 10^−7^ m^2^/s, which was considerably greater than infrared and tray drying (Table 1). Similarly, the activation energy (*E*_a_) is almost twice in FBD (17.33 kJ/mol). Because of the high heat and mass exchange rates, FBD has a rapid drying rate. High heating rate is produced with tightly controlled temperature in the bed; when infrared (IR) was combined with the FBD, the drying rate and quality of the SPs were significantly improved (68). Continuous high-capacity FBD technology is extensively utilized in the pharmaceutical, food, fertilizer, and many other chemical industries.

The potency of using fluidized bed dryers is greatly influenced by energy efficiency. This is expressed as the amount of energy applied to vaporize water from solids (*E*_*w*_) to power provided to the air during drying *(E*_*a*_), with the energy consumed to evaporate moisture calculated as:


(2) $$\begin{array}{l} E_{w}=M_{w}\times L_{w} \end{array}$$

*E*_*w*_ = evaporated water energy (kJ),

*M*_*w*_ = water mass evaporated (kg),

*L*_*w*_= latent heat water vaporization (kJ/kg).

The energy required to heat the air can be calculated as follows:


(3) $$\begin{array}{l} E_{a}=M_{a}\times C_{p}\times \Delta T \end{array}$$

Where,

*E*_*a*_ = the amount of energy used to heat the air (kJ),

*M*_*a*_ = mass of air (kg),

*C*_*p*_ = the air's specific heat (kJ/kg°C),

Δ_*T*_ = temperature difference (°C).

As a result, the thermal dryer efficiency, η_th_, can be calculated as follows:


(4) $$\begin{array}{l} \eta_{th}=\frac{E_{w}}{E_{a}}\times 100\% \end{array}$$

### Oven drying

**Figure 4:**
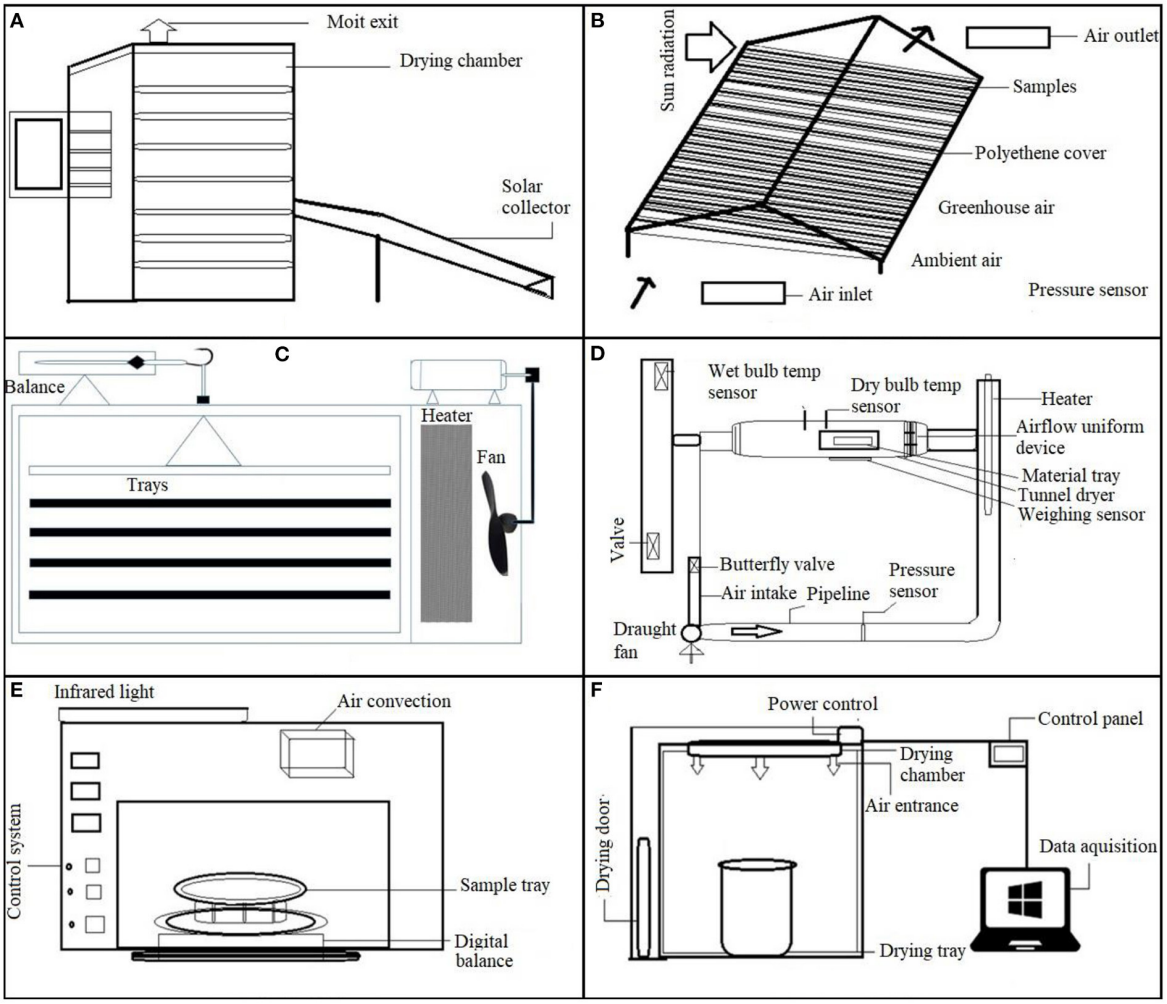
Drying techniques used for sweet potatoes. **(A)** Indirect solar dryer. **(B)** Greenhouse dryer. **(C)** Cabinet dryer. **(D)** Drying system. **(E)** Infrared drying equipment. **(F)** Mid-infrared dryer.

Oven drying (OD) is a simple method for drying food because it does not require any special or extra equipment (Table 1). Its drying rate is faster than that of sun-drying (Figure 4A) and green house dryer (Figure 4B). However, a significant disadvantage is that it can only be done on a limited scale. Since thermal ovens generally feature chamber volumes ranging from 20 to 800 L with a 5–300°C temperature range above ambient temperature. Heat is transmitted to the chamber through the air flow. Antonio et al. (55) reported higher water loss in SPs slices with NaCl and sucrose solution.

Similarly, adding NaCl to the permeable solution increased the driving force of the process, and the dehydration process was verified to be mainly affected by changes in the NaCl concentration. Clifford et al. (54) found similar results: NaCl solution reduced significant water loss in yellow flesh SPs (YFSP), but the salt solution had no significant effect on β-carotene reduction, although degraded β-carotene. Bengtsson et al. (28) studied oven-dried orange-SPs (var. Ejumula) and observed an 11 and 16% loss of β-carotene content during oven drying at 57°C.

The total energy was estimated by Beigi (69) in oven drying using the following equation is:


(5) $$\begin{array}{l} E_{t}=\;E_{th}+E_{mec} \end{array}$$

Where *E*_th_ = sum of thermal energy,

*E*_mec_ = sum of mechanical energy,


(6) $$\begin{array}{l} {\text{E}}_{\text{th}}=\left(A.v.\rho_{\text{a}}.Ca\Delta \text{T}\right).\text{t } \end{array}$$

Where A, v, Δ *and* T are the area of the tray,

(m^2^), airflow rate (m/s), and disparity in temperature (K), respectively.

Also, ρ_a_ = density (kg/m^3^) and C_a_ = specific heat capacity (kJ/kg/K) of inlet air.


(7) $$\begin{array}{l} {\text{E}}_{\text{mec}}=\Delta \rho .m_{air}\cdot t \end{array}$$

ΔP = pressure difference (mbar) and m_air_ =inlet air mass (kg).

### Cabinet drying

Cabinet dryers are the most used in the food industry, as shown in Figure 4C and listed in Table 1. In contrast to oven dryers, which usually increase the temperature of the product to allow preheating or curing, cabinet dryers subject the SPs to 50–80°C temperature for 2.5–24 h. Alternatives to such dryers are tunnel dryers or biomass dryers using charcoal or firewood, which also require fuel investments that lead to poor product quality unless produced sustainably. Despite the drawbacks of cabinet dryers, several studies have been done on cabinet-dried SPs' drying kinetics and nutritional aspects. Kosambo (70) used an electric cabinet drier to dry. Drying fresh slices of 13 orange flesh sweet potatoes (OFSP) cultivars at 58°C for 4 h, resulting in a 35% drop in trans-carotene content. Whereas drying was performed by using cabinet drying and open-air sun drying, losses for SPK004 were found to be 28 and 83%, respectively, and 47 and 72% for Jonathan kinds of SPs samples, respectively. As a result, cabinet drying retained more provitamin A than sun drying.

Similarly, Lohachoompol et al. (71) investigated the effect of cabinet drying on the anthocyanin content of blueberries (*Vaccinium corymbosum* L.) and discovered a 49% loss after drying. However, Joykumar Singh and Pandey (72) studied a different aspect of cabinet drying and observed that the effective moisture diffusivity augmented with rising temperature. Higher forced convection drying reduces heat loss and thus, enhances the dried SPs quality (Figure 4D). The fan or blower propels the drying medium (typically air) through the heater to increase the temperature and lower the RH of the air, thereby improving the heat and mass transfer rate (73). SPs dried at a decreasing rate with an inconstant drying rate during forced convection drying. SPs dried in forced air retained more β-carotene than those dried in open-air (28). To achieve the final moisture level, the drying time of the forced convection tray dryer was maintained at half of that of the free convection dryer by (15). The logarithmic model best fit for the drying data (17). Similarly, Doymaz (64) reported a logarithmic model indicating the best-fit model with the shortest drying time in blanched SPs slices.

The energy efficiency was considered as the ratio of energy used to energy input as below:


(8) $$\begin{array}{l} \eta_{E}=\frac{E_{i}-E_{o}}{E_{i}}=\frac{m_{a}\left(h_{dci@T}-h_{dco@T}\right)}{m_{a}h_{dci@T}}\times 100 \end{array}$$

Where η_*E*_ Is the energy efficiency%, m_a_ is the mass of air (kg s^−1^); *E*_i_ and *E*_o_ are the input and output energies in kJ s^−1^. *h*_*dci@T*_ is the enthalpy of air at the drying chamber's inlet at temperature. *h*_*dco@T*_ is the air enthalpy at the drying chamber's outlet at temperature T.

## Specially modified dryers

### Microwave spouted/assisted bed drying (MSBD)/(MASBD)

**Figure 5:**
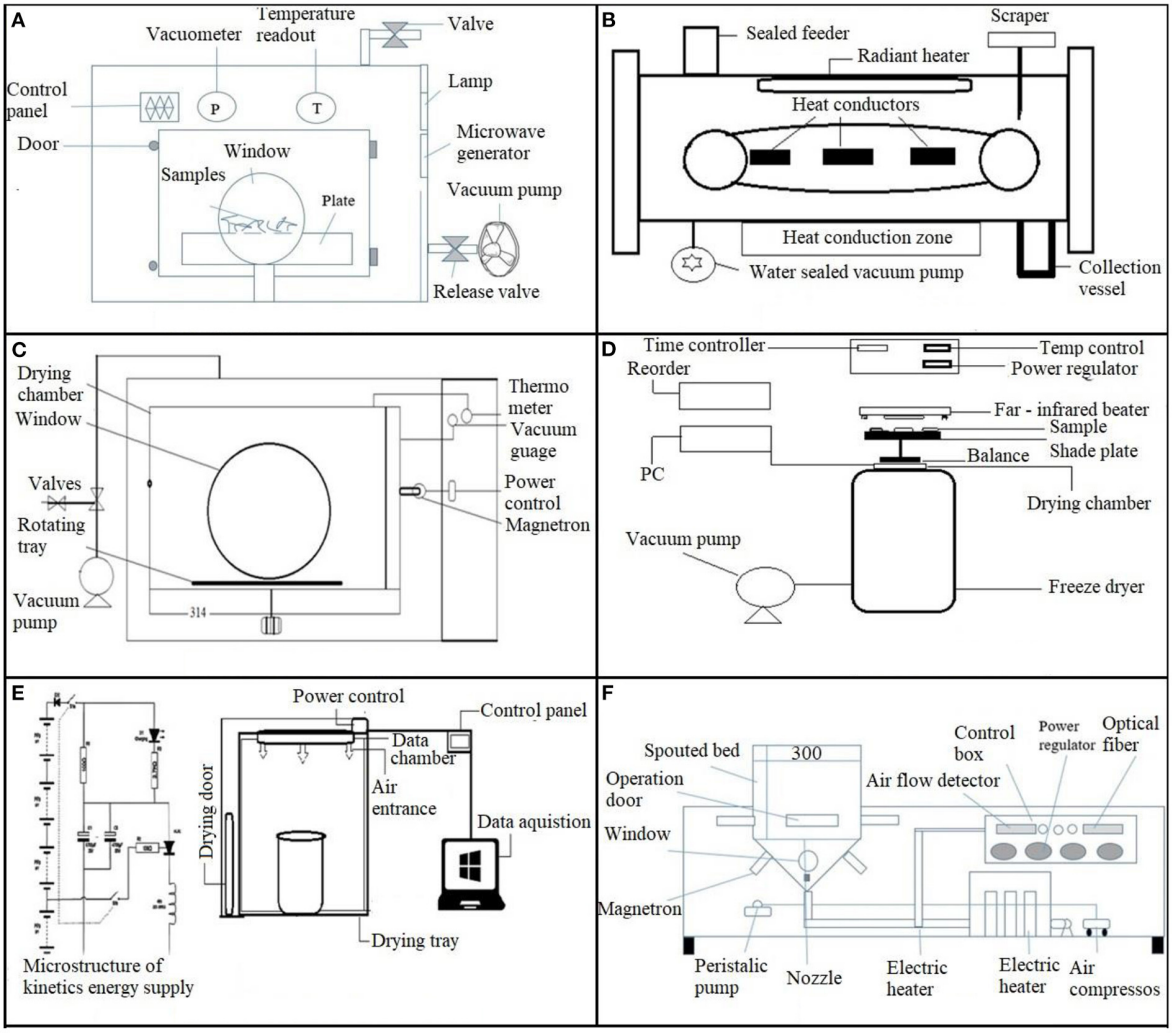
Special purpose drying techniques are used for sweet potatoes. **(A)** Vacuum microwave dryer. **(B)** Continuous vacuum belt dryer. **(C)** Microwave vacuum dryer. **(D)** Freeze dryer and far-infrared dryer. **(E)** Combined hot air and infrared dryer. **(F)** Microwave spouted bed dryer.

More consistent drying can be achieved with a microwave-enhanced spouted bed. Pneumatic agitation in spouted bed dryers allows items to be exposed to microwave energy uniformly (32). Fluidization also enhances mass and heat exchanges because of a constantly replenished boundary layer at the particle surface. As a result, a combined fluidized-spouted bed is an efficient method for resolving the irregular problems of MW drying. These dryers are more effective than standard dryers because of their shorter drying time and better jet velocity to guarantee excellent mixing (32). They have been widely employed in various industrial processes (Table 2). The continual movement of sweet potato cubes within the microwave chamber accounts for the rapid drying rate in MSBD (Figure 5F). Furthermore, the constant movement of the cubes allows various areas of the system to receive relatively homogeneous MW radiation. MSBD-dried sweet potato cubes absorb microwave energy more equally than fluidized samples. Purple flesh sweet potatoes (PFSP) subjected to be dried in a microwave-assisted spouted bed drier (MWSP) had a low rehydration capacity due to the amylose to amylopectin ratio, which is not suited for microwave heating. Due to the rapid movement of products with higher microwave power, products were crispier than those from other driers. A greater microwave power causes fast moisture evaporation during the MSBD process, which causes the formation of porous structures (32). Although steam blanching can help keep color and anthocyanin content, MASBD drying PFSP cubes is generally not an appropriate processing technique, even with the coating treatment (41).

**Table 2:** Hot-air drying combined with other drying methods was conducted to dry sweet potatoes.

**Drying methods**	**Drying conditions/Temperature (°C)/Geometry**	**Size (mm)**	**Response**	**Main conclusion**	**References**
Air/sun/solar drying	HAD, OSD, and STD; *G* = Slices.	–	Provitamin A, carotenoids contents	OSD and STD didn't affect carotenoids. Low-temperature storage decreased provitamin A	(16)
Air drying/freeze drying	OD = 30, 70 and 100°C; FD = −20°C, *G* = leaves; *V* = 1.5 m/s	–	CQA derivatives and antioxidants	Freeze-drying preserves CQA and antioxidants, while 70 and 100°C drying preserve both of them	(24)
Air drying/microwave/ vacuum	OD = 65°C, 9 h, *P* = (800 W, 50 Hz), 5 min, FD = −50°C, 36 h; *G* = Slices	5	Antioxidants, phenols, ascorbic acid	Microwave drying increased TPC and antioxidants. β-carotene reduced sweet potato slice's antioxidant activity.	(25)
Spray drying	Inlet *T* = 200°C; Outlet *T* = 100°C; *G* = Puree	–	Physicochemical, antioxidants	MD flour had a high antioxidant and retained anthocyanins, total phenols, and flavonoids.	(26)
Sun drying	OSD; *T* = 54°C; *T* = Slices	1.5	Protein content, fiber, moisture, β-carotene	Matobolwa sweet potato retained more β-carotene than michembe variety after 6 months' storage	(27)
Air/Sun/Solar tunnel drying	*T*= 57°C, STD *T* = 45–63°C, OSD *T* = 30–52°C	1–2	Carotenoid profile, β-carotene, color	Forced-air oven drying, STD, and OSD reduced all-trans β-carotene by 12, 9 and 16%	(28)
Air drying/IR radiations	HAD *T*= 50°C, 55 and 60°C, IR *T* = 250 W, HP *T* = 180°C; *G* = chips	6, 8	Drying kinetics, sugar, taste, texture	IR radiations assisted thick sweet potatoes at 60°C. IR radiation at 60°C for 5 h and HP drying was suitable for mass-producing sweet potato snacks	(29)
Spray drying	*T* = 85°C; *G* = slices	2-3	Phytochemicals, antioxidants, color, microstructure	Encapsulated flour had higher TPC, antioxidant, and water solubility than non-encapsulated flour	(30)
Intermittent IR and convective drying	*IR*= 1,100 and 1,400 W/m^2^; *G* = slices	4, 36	Mathematical simulations, color, microstructure	IR drying is more effective than convective hot air drying for product quality	(31)
Air/Microwave-vacuum drying.	SBD, HAD = 80°C; MVP = 2.0 W/g; VP = 5 kPa; *G* = dices	10 × 10 × 10	MC, rehydration ratio, crisp degree, expansion ratio	Microwave-spouted bed and MVP showed faster drying, better rehydration, uniform color, and high β-carotene retention than HAD	(32)
IR drying	*P* = 104, 125, 146, 167 W; *G* = slices	3, 5, 8	Drying kinetics, rehydration ratio	Increasing power reduced drying time. IR affects RR and D_eff_. The log model fits drying curves	(33)
Air drying/IR/Combine IR and air drying	HAD = 50, 60 and 70°C; *IR* = 1,100, 1,400 (W/m^2^); *G* = slices	4, 5	Drying kinetics, energy consumption, E_a_, color, microstructure	Combined IR and HAD drying had the shortest drying time, lowest energy consumption, and best color attributes	(34)
Sun/Tent/tunnel drying	*T* = 10, 20, 30, 40°C; *G* = slices	2	Carotenoids, dry matter, drying kinetics	OSD decreased carotenoids less than tent and tunnel drying	(35)
Air/tray/infrared drying	FBD = 45, 55, 65°C; tray = 3,000 W; IR; 1,000 W; *G* = pieces	–	Mathematical modeling, starch properties	Midilli model best explains starch drying. The drying affected starch's color, solubility, and gel texture	(36)
HAD/IR drying	HAD = 50, 60, 70; *V* = 1.16 m/s; *RH* = 45°C; IR = 1,100, 1,400 W/m^2;^ *G* = Slices	4–6	Mathematical modeling, *E_*a*_*, color	The two-term model explains sweet potato drying kinetics. *E_*a*_* = 12.83 to 34.64 KJ/mol	(37)
IR-hot air drying	IR-HAD = 60°C; *G* = slices	4	Heat and mass transfer	Lambert's law explains sweet potato heat and mass transfer	(38)
IR heating	IR = 1,400 W/m^2^; *G* = slices	4	Heat and mass transfer, shrinkage	Mass and heat transfer coefficients, shrinkage, and IR heating affected moisture distribution during drying	(31)
Tunnel/shade/open-air drying	*G* = chips	–	Carotenoid contents	All dryers lost 9.2% carotenoid. After 4 months of storage, the carotenoid loss was 83%	(39)
Sun drying	OSD, 10 a.m. to 4 p.m.; *G* = leaves	–	Proximate analysis, anti-nutrient content	Purple midrib sweet potato leaves had more fiber, ash, carotenoids, iron, calcium, and polyphenols	(40)
Microwave drying	MSBD = 60°C; Steamed = 100°C; *G* = Cubes	10 × 10	Rehydration, color, texture, anthocyanins	Steamed coating with sodium alginate reduced drying time, improved color, and reduced rehydration	(41)
Vacuum drying	VD = 100°C, 120°C, and 140°C; *P* = 2.67 kPa; *G* = Chips	0.80, 1.50	Drying kinetics, β-carotene, color, texture	High-likeability mix temperatures maintain color and -carotene	(42)
IR/Air drying	IR = 1,100 W/m^2^, HAD = 50–70°C; *G* = Slices	4	Drying, phytochemicals, color	The two-term model fits best, and IR-HAD was the best method	(43)

HAD, hot air dryer/drying; OSD, open sun drying; STD, solar tunnel dryer/drying; OD, oven dryer/drying; FD, freeze drying; IR, infrared; MVP, microwave-vacuum drying.

The drying process's energy efficiency (η_*e*_) is described by,


(9) $$\begin{array}{l} \eta_{e}\\ =\frac{W_{d}\left[h_{fg}\left(M_{p1}-M_{p2}\right)+C_{m}\left(T_{m2}-T_{m1}\right)+m_{fw}\left(h_{fw2}-h_{fw1}\right)\right]}{m_{da}\left(h_{1}-h_{o}\right)\Delta t+\Delta tQ_{MW}} \end{array}$$

Where W_d_ weight of dry material (kg); c_m_ = material specific heat (kJ/kg K); h_fg_ = is the latent heat of vaporization, M_p_ = dry based particle moisture level (kg _water_/kg _solid_); P_1_ microwave power density immersed by a dielectric material (kW/cm^3^); P_2_ energy is needed to heat material (kW); Q_MW_=microwave energy (kW).

### Microwave-assisted freeze-drying

Microwave heating replaces traditional conduction heating during freeze-drying, allowing the benefits of both microwave drying and freeze-drying to be combined in a process known as microwave-freeze drying. The combined freeze-drying and microwave energy use is microwave-assisted freeze-drying (MWFD) (Table 2). The microwave drying system adjusts the control sample temperature, sample mass, and microwave power (74). Huang et al. (75) revealed that MWFD chips had the best quality, were preferred by customers, and had a shorter drying time than FD chips in restructured mixed potato with apple chips. In addition, microwave energy increases the drying rate, final product quality, and energy consumption. A study by Liu et al. (41) demonstrated that MWFD is a time-consuming process for drying SPs, and energy consumption was twice as high as in microwave vacuum drying (MWVD) (Figure 5C). However, MWFD could preserve better anthocyanins, and sensory evaluation was remarkable concerning the crispiness of SPs. The potential of this drying technique requires further exploration of SPs.

An electric energy meter can calculate the energy consumption of the three parts during drying: the vacuum system, cold trap, and heating system.


(10) $$\begin{array}{l} ES=\frac{E}{M} \end{array}$$

Where ES denotes specific energy consumption (kJ/kg), E denotes total energy consumption (kJ), and M represents the weight of moisture reduced from the samples (kg).

### Microwave vacuum drying

It is a new technology that operates microwave radiation as a heat source in a sub-climatic pressure environment (Figure 5A) and exhibits the benefits of both microwave and vacuum drying. Likewise, sublimation drying, vacuum and microwave energy's instantaneous and direct volume heating, and low drying temperature can both increase energy efficiency and quality of products (76). Marzuki et al. (77) revealed that PFSP dried in 6–12 mins under microwave vacuum drying (MVD) conditions, significantly faster than hot-air drying (600 mins) and total phenolic content (TPC), color, and antioxidant activity improved. Similar findings were described by Lagnika et al. (78), who found that anthocyanins and total phenolics were abundant in PFSP. Correspondingly, total carotenoid and vitamin-C levels were abundant in OFSP. As microwave vacuum drying produces excellent nutritional and sensory quality food with slight shrinkage. Monteiro et al. (79) found that MVD is an appropriate procedure for making highly porous sweet potato chips, enhancing the value and prolonging the shelf life of vegetables. Furthermore, research on the bioactive components of purple and orange sweet potato slices impacted by MVD after pretreatment is restricted. This knowledge gap may affect the efficient production of premium products from these two sweet potato varieties.

The following equation can calculate microwave-vacuum drying's energy efficiency:


(11) $$\begin{array}{l} DF=\frac{t_{on}p\left(1-m_{f}\right)10^{-6}}{M_{i}\left(m_{i}-m_{f}\right)} \end{array}$$

Where t_on_ (s) = microwave drying exposure time at the applied power input, P (W), Mi = the sample's initial mass (kg). The initial and final moisture levels are m*i* (kg) and m_f_(kg), respectively.

### Catalytic infrared drying

The catalytic infrared drying (CIR) emitter is motorized by propane or petroleum gas to generate thermal radiant energy *via* a synergistic reaction with a catalyst pad inside the CIR transmitter (Figures 4E,F) (Table 3). The CIR emitter uses less energy than traditional infrared emitters, which use electricity to convert natural gas straight into radiant radiation (80). Manu et al. (81) used a flameless gas infrared catalytic drier to dry mango-sweet potato leather with a moisture content of 15.4% at 45, 50, and 55°C. The cabinet, oven, and solar-dried jack fruit leather moisture levels were 18.85, 14.79, and 18.5%, respectively (82). They determined that a catalytic infrared drier was more feasible in drying mango-sweet potato leather than a cabinet, oven, or sun dryer. Similarly, Rashid et al. (83) noted a faster drying rate in a CIR drier at 20 and 40 kHz US frequencies at 70°C compared to HAD at 60, 70, and 80°C for SPs dried by Onwude et al. (37).

**Table 3:** Application of different pretreatments along with varying methods of drying on drying of sweet potatoes.

**Drying methods**	**Drying conditions/ Temperatures (°C)/Geometry**	**Pretreatments**	**Size (mm)**	**Response**	**Main conclusion**	**References**
Catalytic infrared drying	IR *T* = 60, 70, and 80°C; *G* = Slices	Multi-frequency US drying (20, 40 and 60 kHz)	3	FTIR, SEM, phytochemicals, drying kinetics	40 kHz at 70°C reduced drying time. Ellagic acid and Rutin were higher, and 20 kHz. FTIR showed the OH group and phenolics	(44)
Microwave drying	*P* = 700-W; *G* = Circular slices	Carbonic maceration (CM)	5	Drying, physicochemical, and antioxidant properties	Intermittently dried CM has high phytochemical and antioxidant activity, reduces drying time, and lowers *E_*a*_* (DPPH)	(45)
Freeze drying	FD = 13.3 Pa; *G* = Cubes	Heat treatment after high-pressure treatment	20 × 20 × 10	Rehydration, color, rheological properties	Pretreatments did not affect color or gelatinization rate but improved texture	(46)
HAD	HAD+US; *T* = 40, 50, 60, 70°C; *G* = Slices	Drying kinetics, moisture diffusion, energy consumption	5	Ratio and reduce energy consumption	US power increases reduce drying time, color difference, and rehydration	(47)
Contact US-HAD at 40°C	US-HAD; *T* = 40°C; *G* = Slices	Hyperspectral imaging, anthocyanins	5	–	RC-MLR predicts anthocyanins best	(48)
Natural drying	OSD, STD; G = Slices	Blanching with boiling water	1.50	β-Carotene, mineral content	Fresh samples had less-Carotene and more fat, protein, fiber, and carbohydrates	(27)
Air drying	HAD; *T* = 50–80°C; *G* = Slices	Boiled water blanching, slices	5, 10, 15	Drying kinetics, mathematical modeling, E_a_	Page and modified were the best models; *E_*a*_* was 11.1–30.4 KJ/mol	(18)
Ai drying	HAD; *T* = 50–80°C; *V* = 1.25 m/s; *G* = Slices	Boiled water and metabisulphite blanching, slices	4	Drying kinetics, mathematical modeling	The modified page model was the best. Boiling water and metabisulphite improved drying over control	(49)
Air drying	HAD; *T* = 55–65°C; *G* = Flour	Sodium hydrogen sulfite (NaHSO_3_)	1	Phytochemicals, color, SEM	Pretreated flour showed higher phytochemicals and color change than control	(50)
Air drying	HAD; *T* = 55–65°C; *G* = slices	Citric acid pretreatment	1	β-Carotene, ascorbic acid, TPC	β-carotene, TPC, and ascorbic acid values were close to predictions	(51)
Sun drying	OSD; *G* = Slices, chips	Sodium metabisulphite, ascorbic acid, citric acid, and salt	–	Total carotenoids, storage of sweet potatoes	Ascorbic acid, sodium metabisulphite, citric acid, and salt improved carotenoids in the first month of storage but not after 4–6 months	(52)

HAD, hot air dryer/drying; CD, cabinet dryer; TD, tray dryer/drying; OD, oven dryer/drying; DD, drum dryer/drying; US, ultrasound; D_eff_, moisture effective diffusion/moisture diffusivity; E_a_, activation energy; SEM, scanning electron microscopy; TPC, total phenolic content; KMS, potassium metabisulfite; FTIR, Fourier transform infrared spectroscopy; POD, peroxidase enzyme.

The energy efficiency calculated by Coskun et al. (84) of an infrared dryer can be calculated below.


(12) $$\begin{array}{l} E_{T}=R_{il}+E_{cb} \end{array}$$

where E_il_ is the energy consumed by infrared lamps and *E*_cb_ is the energy used by the centrifugal blower.


(13) $$\begin{array}{l} E_{il}=k\times t \end{array}$$

k represents lamp power, and t represents drying time.


(14) $$\begin{array}{l} E_{cb}=\left(\frac{V^{3}}{16600}\right)\times t \end{array}$$

V represents the air velocity (m/s).

### Combine hot-air and infrared drying

Traditional drying processes are widely utilized in agricultural commodities to preserve and process agricultural goods. In particular, hot air drying (HAD) is extensively employed for commercial and industrial food preparation. It consumes a lot of energy and may impact the final product quality due to a longer drying time (38, 64). As a result, novel and inventive drying procedures have attracted considerable attention. It has been revealed that combining infrared and hot air-drying techniques (Figure 5E; Table 2) can increase energy efficiency and dried agricultural product's quality. However, research on its applicability to drying commercial crops, such as SPs, is limited because it is a new drying process. The only study conducted by Onwude et al. (37) reported that combining IR and HAD techniques resulted in shorter drying times and lower energy consumption than HAD and IR dryers. Meanwhile, different combinations of IR + HAD substantially influenced the phytochemical characteristics of dried SPs, resulting in improvements in TFC, TPC, and DPPH. Overall, the combination of IR and HAD showed significant potential, providing more valuable knowledge of the sweet potato drying process than traditional dryers.

Energy efficiency is calculated by dividing the energy required for moisture evaporation from the drying product by the total energy SEC consumed during the drying process.


(15) $$\begin{array}{l} \eta_{e}=\left(\frac{E_{evap}}{SEC}\right)\times 100 \end{array}$$

where η_*e*_ denotes energy efficiency, and *E*_evap_ indicates the amount of energy required to evaporate moisture (kJ).

### Continuous vacuum belt dryer/vacuum freeze-drying

A continuous vacuum belt drying system (Table 2) was developed so that the product moves along the belt under a vacuum and is heated by conduction or radiation (Figure 5B). It has been used to commercialize instant tea, high-quality citrus crystals, and medicines. The only study by Xu et al. (42) examined the application of comparatively low-temperature vacuum belt drying to eliminate water without significantly changing the phytonutrients of vegetables while creating a crisp structure. Crispy sweet potato chips with nice color preserve a significant amount of β-carotene and are preferred by customers can be produced *via* vacuum-belt drying at 100–120°C or a specified temperature amalgamation.

The drying efficiency is the amount of heat energy used to evaporate moisture from fruits to the total energy consumed.


(16) $$\begin{array}{l} \eta =\frac{M_{w}.L}{E_{T}} \end{array}$$

Where MW = mass of water evaporated in kg, and *L* = latent heat of vaporization in KJ/kg.

**Figure 6:**
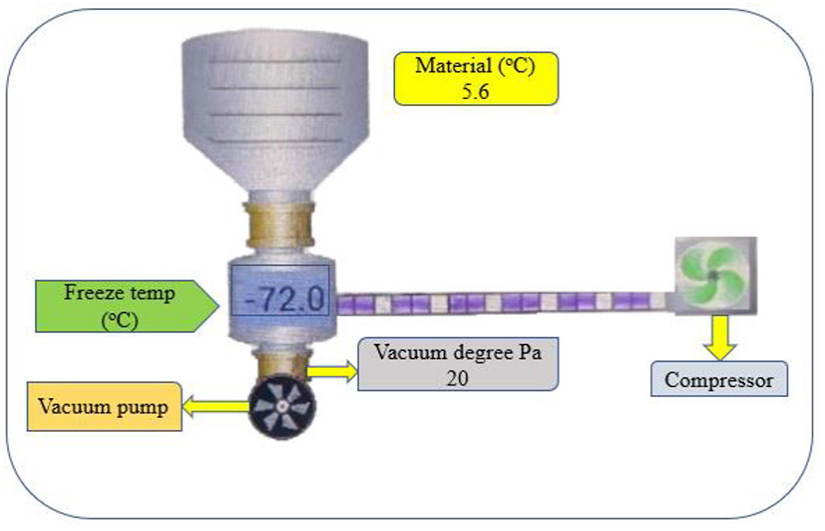
The mechanism of vacuum freeze drying.

Vacuum freeze-drying (VFD) is a technique for solidified materials drying by water sublimation under a vacuum (Figure 6; Table 2). It is the best method for removing water from the final product in comparison with other drying techniques (85). VFD highly preserved β-carotene in SPs compared to microwave and hot-air drying (HAD), but VFD and HAD require days to dry samples, whereas microwaves can dry in only minutes. Yang et al. (85) further noted that microwave and hot air drying provided more phenolics and antioxidants. Similarly, the drying time in VFD improved the brightness of purple SPs but had little effect on other color variables (86). Deng and Jiang (87) concluded that VFD has the lowest comprehensive effect on sweet potato flour than HAD and vacuum drying (Figure 5D). Therefore, VFD is the most cost-effective method of obtaining sweet potato flour and requires further exploration.

The energy efficiency of vacuum drying can be calculated as below,


(17) $$\begin{array}{l} E_{T}=E_{h}+E_{r}+E_{c}+E_{v}+E_{s} \end{array}$$

*E*_h_ = denotes hydraulic energy.

*E*_r_ = stands for refrigeration energy.

*E*_c_ = stands for circulatory energy.

*E*_v_ = is the vacuum's energy.

*E*_s_ = stands for energy for sublimation.

## Drying pretreatments

To reduce nutritional loss and enhance the quality of dried food materials, pretreatments are commonly used with the drying method. The effects of chemical and osmosis pretreatment on dry SPs' nutritional and bioactive properties are summarized below and in Table 3.

### Chemical pretreatments

These can significantly improve drying kinetics; however, they can also result in the loss of soluble nutrients and chemical residues, producing food safety issues.

Citric acid is an organic acid used in fruits and vegetables as a texture modifier for anti-browning treatments. Meanwhile, citric acid has been shown to speed up drying because pectin loosens in an acidic environment, allowing water to be removed (88). Table 1 highlights the effect of CA pretreatment on sweet potato color retention and drying rate. Singh et al. (63) discovered that pieces of sweet potato treated with 1% CA at 50°C had improved color and required less energy to dry. Another study found that pieces treated with KMS (1.0%) (Potassium Metabisulfite) and CA (1%) at 50°C had improved color with less energy to dry (89). On the other hand, treatment with 1% CA preserved fewer bioactive compounds and antioxidants after drying at 55°C than 3% CA treatment, showing that 3% CA pretreatment resulted in better preservation effects (90).

Sulfidation, often known as sulfuring, is a standard technique for reducing obscuring throughout drying and maintaining quality during food processing and storage on an industrial scale (25). Commonly utilized sulfur dioxide gas or water-soluble sulfide salts include potassium metabisulfite (K_2_S_2_O_5_), sodium metabisulfite (Na_2_S_2_O_5_), and sodium hydrogen sulfite (NaHSO_3_). The drying methods (hot-air and drum dryer) increased the anthocyanin content of SPs by 1.8 to 3.8 times by pretreatment with (0.5% w/v) sodium metabisulfite; however, the drying process resulted in a considerable loss of β-carotene. Compared to hot-air drying, drum drying produces sweet potato flour (SPF) with superior color, TPC, and antioxidant activity (91). Desulfurization treatment can improve the quality of SPs, such as rehydration ratio, β-carotene content, and color (35, 63). The quality improvements in sweet potato chips after potassium metabisulfite and sodium chloride treatment (92). The flour quality of OFSP was also increased by sodium hydrogen sulfite solution (50).

The carbonic maceration (CM) strategy is used in its general application. It involves placing samples in a carbon dioxide-rich sealed tank an adaptation that is immediately reflected in the transition of plant internal materials from respiratory to fermentative anaerobic digestion (93). Mainly, pretreatments of CM have been used in drying chili and raisins (94, 95), respectively. However, only one study explores the impact of CM pretreatment on sweet potato drying behavior. SPs were processed with CM, which upgraded the drying procedure and improved the dried good quality. CM pretreatment reduced sweet potato drying time by 38.1–34.6%, and the phytochemicals (phenols, anthocyanin, flavonoids, -carotene substance, and ascorbic acid) and DPPH radical activity was 13.83–78.18 and 10.04–14.09% higher than those of untreated samples, respectively (45).

NaCl is used as an antioxidant, improving sulfurous acid's antioxidative activity. NaCl also restrains the movement of oxidizing catalysts, for example, the polyphenol involved in peeling and cutting fruits and vegetables with discoloration (96). Osmotic solutions, such as salt, mainly reduce SPs' drying time and product quality. The same trend was noted by Singh et al. (92) for the quality enhancement of sweet potato chips using NaCl as an osmotic agent. On the other hand, the salt solution had no leaching effect on β-carotene and acted as an antioxidant agent for β-carotene in SPs (54). On other aspects of SPs in a NaCl solution and found that the salt solution reduced the drying time and effective diffusivity according to Fick's equation diverse from 3.82 × 10^−10^ to 7.46 × 10^−10^ m^2^/s for water and 1.18 × 10^−9^ to 3.38 × 10^−10^ m^2^/s for solids (55). Similar findings were confirmed by Clifford et al. (54), who reported that salt solution could be an energy-saving cost for the dehydration of SPs.

### Osmotic pretreatments

**Figure 7:**
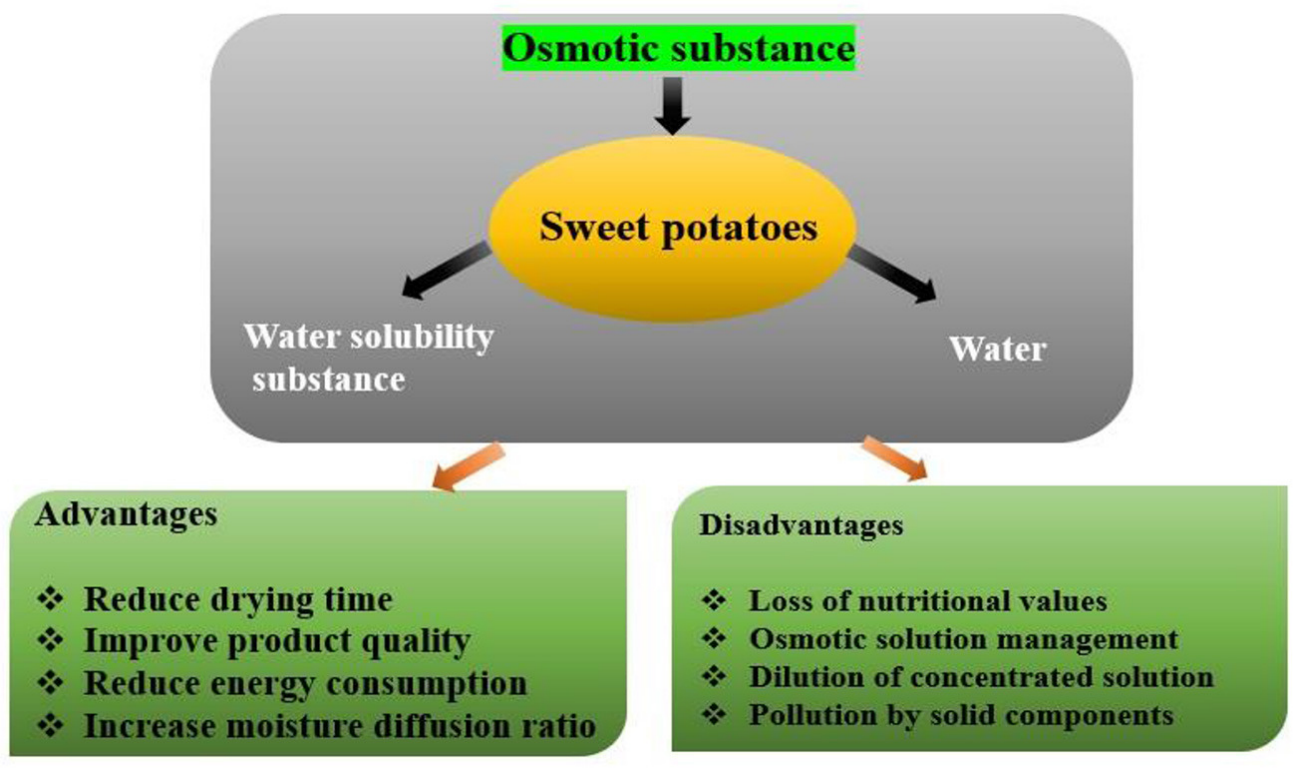
Osmotic dehydration principle of sweet potato drying.

Osmotic dehydration removes water from fresh food by immersing it in a solution with high permeation pressure, which has less water activity (Table 4). As demonstrated in Figure 7, during osmotic pretreatments, the cellular structure works as a semi-permeable barrier, allowing for countercurrent mass transfer. As the solute enters the products, internal moisture is transferred to the hypertonic solution (97). Researchers have compared different types of osmotic solutions, such as sucrose, glucose, fructose, and sorbitol (54, 56, 57, 89, 98–103).

**Table 4:** Application of different osmotic solutions as pretreatments along with varying methods of drying for optimizing drying protocols for sweet potatoes.

**Drying methods**	**Drying conditions/ Temperature (°C)/Geometry**	**Osmotic solution**	**Size (mm)**	**Response**	**Main conclusion**	**References**
Air drying	HAD; *T* = 30–60°C; *G* = Slices	Sugar solution 40, 50, and 60%	3, 5	Water loss (WL), solid gain (SG)	At 60°C, a 60% sugar solution had the best WL and gain. WL and SG were highest in controls.	(53)
Air drying	*T* = 60°C; OD = 10–15% w/v; *G* = Slices	NaCl solution	2,3,4	β-carotene, *D_*eff*_*	Salt did not affect β-carotene degradation, but OD decreased.	(54)
Air drying	OD = 0, 5, 10% w/w; *G* = Slices	Sucrose, sorbitol, fructose	20 × 20 × 5	WL, SG, water activity	Sorbitol's WL was higher than fructose's SG. Barbosa Junior showed the best fit model	(55)
Air drying	OD = 40% sucrose, 5% w/w salt; VD = 70°C; *G* = Slices	Sucrose, NaCl	0.5	WL, SG	Azuara model best fit. WL and SG of sweet potatoes increased with time	(56)
Ultrasound microwave vacuum drying (MVD)	HAD; *T* = 65°C; OD = 35°Bx; MVP = 22 W/g; P = 90 KPa; *G* = Slices	Sucrose solution	10	WL, SG, expansion ratio, rehydration ratio, color, texture, SEM	The US rehydrated more. The osmotic group improved WL, and SG improved color, taste, and texture. Osmotic and US drying improved	(57)
Microwave drying	*P* = 180 and 350 W; OD = 1% w/v; *G* = Slices	Sucrose, fructose, sorbitol	20	Drying kinetics, mathematical modeling	Weibull fits best. OD and microwave power reduce drying time	(58)

HAD, hot air dryer/drying; CD, cabinet dryer; TD, tray dryer/drying; OD, oven dryer/drying; DD, drum dryer/drying; US, ultrasound; D_eff_, moisture effective diffusion/moisture diffusivity; E_a_, activation energy; SEM, scanning electron microscopy; TPC, total phenolic content; WL, water loss; SG, solid gain; HTST, high-temperature short time.

Sucrose is the most often used osmotic agent in sugar pretreatments due to its low cost and high mass transfer (104). Adding sucrose to a product improves the dried product's sweetness and calorie content. Substances e.g., fructose and sorbitol may be utilized to minimize water activity. Fruit-derived agents have smaller molecular weights than sucrose, a sweet flavor, and lesser calories (105–107). They inhibit enzymatic and non-enzymatic browning activities, reducing energy consumption while preserving color and smell. They also remove surface and intercellular gases, which prevent oxidation, browning, softness, and off-flavor formation. Most sweet potato OD studies used sucrose and NaCl as osmotic agents (55). There have been few studies on the nutritional aspects, shrinkage, and drying kinetics that occur throughout the process and non-ionic agents like sorbitol, glucose, and fructose for the OD of sweet potato. One of the studies by Brochier et al. (106) observed that OD (sucrose, sorbitol, fructose) was an excellent pretreatment for microwave drying of SPs with a shorter drying time. Weibull models gave a best-fit model for microwave drying.

Similarly, de Junqueira et al. (89) found sorbitol as the finest osmotic agent, with the most significant water loss and the minimum gain of solid. Fructose was more efficient at reducing water activity (a_w)_ in the samples, although it resulted in more solid absorption. The absolute sugar concentrations of the SPs studied ranged from 4.8 to 12.5%, with glucose, fructose, and sucrose levels in fresh roots varying between genotypes (108).

### Ultrasound pretreatments

**Figure 8:**
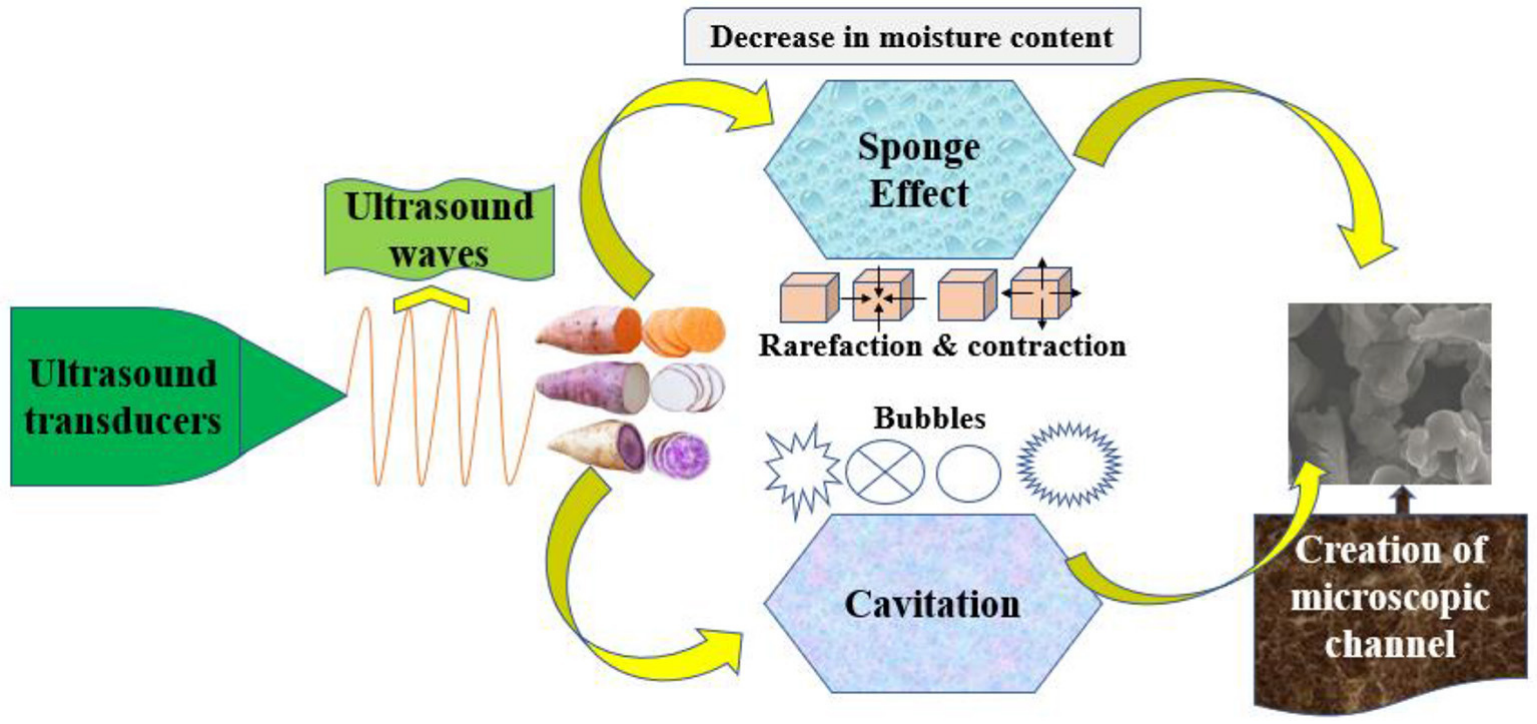
Ultrasound impact on sweet potatoes samples.

Ultrasound (US) is a developing technology in the food industry because it has many advantages over traditional food processing methods (Table 4). The sponge effect was also induced by ultrasound, which created a fast alternate compression and expansion of the food matrix. Sonication also generates cavitation in a liquid medium, forming bubbles that can explode and cause confined pressure and temperature expansion (Figure 8). Compared to untreated samples, US pretreatment might enhance the β-carotene concentration by 4–42%. As a result, pretreatments developed in the US can increase the quality of SPs though lowering the drying period (109). Tayyab Rashid et al. (1) conducted a series of studies on the ultrasonic drying of SPs. They found that vitamin C was better maintained in the combination ultrasound/glucose treatment, while antioxidant assays were more significant in ultrasound- and glucose-pretreated samples. The effective moisture diffusivity was raised by conducting ultrasound pretreatment to minimize drying duration.

In contrast, according to the findings of ultrasonic-osmotic dehydration, the osmotic solution did not influence moisture diffusivity. Among the various US pretreatment timings, the 30 min ultrasonically osmotic treatment (US/GC-10%-3) was the most effective drying time reduction (100). In another study of multi-frequency ultrasound with infrared drying, an optimized US frequency (40 kHz) at 70°C reduced the drying time to 20 kHz, resulting in higher phytochemical quality in dried samples than in fresh samples (101). Similarly, pretreatment with ultrasound at 40 kHz (70°C) retained the phytochemicals in dried SPs. According to HPLC analysis, the most common phenolic acids were ellagic and chlorogenic (44). The ultrasound treatment had a great impact on the morphology of sweet potatoes for its cavitation and turbulence effect. Lv et al. (110) also reported that generated debris and irregular pores of the ultrasound-treated egg white sample were due to ultrasonic cavitation and the mechanical effect. This led to an increase in water loss and solid gain and the more the water loss and solid gain, the more collapse the cell structure (111). This finding is similar to those reported on egg white by using ultrasound (112). The ultrasound-treated sweet potato samples considerably reduced drying duration from 110 to 60 min in contrast with the control samples. Amongst the 13 explored mathematical models, the Hii, Page, and Silva models adequately reflected drying kinetics (1).

## The effect of drying techniques on sweet potato nutritional qualities

Drying can dramatically change food products' phytochemical properties and other quality attributes. The main characteristics of SPs that must be considered when drying are their proximate composition (fat, fiber, carbohydrate, ash, and protein), vitamins, minerals, anti-nutrients, sensory characteristics, and antioxidant properties (phenols, flavonols, ABTS, DPPH). In general, drying increases the nutritional value (83).

Drying methods, time, and temperature significantly affect SPs' nutritional value. Fat, vitamin C, protein, total carotenoids, and beta-carotene decrease with drying temperatures, while mineral, fiber, ash, and carbohydrate content increase significantly (50, 113). Studies on the proximate composition of SPs revealed that fresh samples had substantially lower proximate composition (protein, fat, fiber, and carbohydrate) and mineral content compared to dried samples because of the significant amount of moisture loss during drying, leading to a rise in the level of other nutrients (113–115). Sun-drying (40, 114, 116) and oven drying (50, 115) are the best methods for preserving the quality of dried SPs. Because of their short drying times, infrared and microwave drying was the most cost-effective of all drying technologies. Onwude et al. (37) revealed that average SEC values obtained were lesser than those acquired for other fruits dried by various drying techniques e.g., convective hot-air drying of mushrooms (47.88–93.45 kW h/kg) (117), vacuum drying of mushrooms (41.97–124.34 kW h/kg) (117), and combined microwave and hot-air drying of longan (8.23–10.08 kW h/kg). Under quicker drying conditions, the qualitative characteristics of SPs used for food were better retained.

Minerals and vitamins are essential nutrients required by our body to function correctly. As with the approximate ingredients, minerals and vitamins significantly affect the drying time, drying method, and drying temperature. Kosambo (70) stated that cabinet drying usually retains more ascorbic acid than sun drying. The loss of trans-carotene (Provitamin A) during sun-drying was higher than that of cabinet drying in SPs. There is little difference between the preservation of vitamin A in the tunnel and the open-air solar dryer of SPs (13 and 10%, respectively) (16). According to Bechoff (35), hot-air crossflow drying preserves more vitamin A than sun-drying. In addition to magnesium, the temperature positively impacts the mineral quality of SPs. The discrete mineral composition of SP flour has seldom been deliberated, whereas the amount of ash is usually reported as the estimated total mineral content. Results from Olatunde et al. (118) indicate that sweet potato roots are a good source of minerals, particularly essential micronutrients like Cu, Zn, iron, and Mn.

## Mathematical models used in drying sweet potatoes

The drying process modeling is a critical component of drying technology, particularly in industrial operations. The essential characteristics of thin layer drying technology are the mathematical modeling of the drying procedure and the design of equipment to select the most suitable operating conditions. These models are often used to describe dried SPs. The developed model has been used for calculations, including designing, and constructing new drying systems, optimizing the drying techniques, and describing the drying behavior, including combined macroscopic-microscopic media for mass and heat transmission. Drying conditions, dryer type, and the material's properties to be dried all affect drying kinetics. These actions speed up the drying process since the material is completely subjected to hot air and temperature drying conditions. These models account for exterior resistance to the moisture transport method between the atmosphere and material, providing a greater degree of accuracy, better predicting the behavior of the drying process, and making fewer assumptions as they rely on experimental information. Therefore, these models have proven most valuable to dryer engineers and designers (11). Though, they are only efficient under particular dry circumstances. Alternatively, the theoretical models include numerous hypotheses, leading to many errors and restricting their use in dryer design (18). To fit the drying data, various drying models were applied. The model parameters k, n, and a were determined by fitting the curve. The determination coefficient was utilized to evaluate the experimental data's fit (R^2^). The model with the least possible RMSE and chi-square χ^2^ and the uppermost R^2^ was chosen as the best fit for sweet potato thin layer drying characteristics. The better the fit, the greater the R^2^ value. Generally, an R^2^ value of 0.97 or above is considered an excellent match (36, 37). Table 5 summarizes the mathematical models suitable for drying various SPs.

**Table 5:** Thin layer models were reported in previous studies for sweet potato drying.

**S. no**.	**Model name**	**Model**	**References**
1	Newton	*Mr* = exp(−*kt*)	(119)
2	Page	*Mr* = exp(−*kt*^*n*^)	(120)
3	Modified page 1	*Mr* = *a*exp(−*kt*^*n*^)	(23)
4	Handerson and Pabis	*Mr* = *aexp*(−*kt*^*n*^)	(121)
5	Modified Henderson and Pabis	*Mr* = *aexp*(−*kt*)+*bexp*(−*gt*)+*cexp*(−*ht*)	(122)
6	Logarithmic	*Mr* = *aexp*(−*kt*)+*c*	(123)
7	Midilli	*Mr* = *aexp*(−*kt*)+*bt*	(124)
8	Two-term	*Mr* = *aexp*(−*k*_1_*t*)+*bexp*(−*k*_2_*t*)	(125)
9	Two-term exponential	*Mr* = *aexp*(−*kt*) + (1 − *a*)exp(−*kat*)	(36)
10	Hii	$Mr=aexp\left({-k}_{1}t^{n}\right)+bexp\left({-k}_{2}t^{n}\right)$	(126)
11	Verma	*Mr* = *aexp*(−*kt*) + (1 − *a*)exp(−*gt*)	(127)
12	Modified Midilli	*Mr* = *aexp*(−*kt*)+*b*	(128)
13	Aghbashlo	*Mr* = exp(*k*_1_*t*/1 + *k*_2_*t*)	(129)
14	Wang and Singh	*Mr* = 1 + *at* + *bt*^2^	(130)
15	Silva	$Mr=\exp\left(-at-b\sqrt{t}\right)$	(131)
16	Jena and Das	$Mr=aexp\left(-kt+bt^{\frac{1}{2}}\right)+C$	(132)
17	Parabolic	*Mr* = *a* + *bt* + *ct*^2^	(133)
18	Weibull model	*Mr* = *a* − *b* exp(−*kt*^*n*^)	(134)
19	Approximation of diffusion	*Mr* = *aexp*(−*kt*) + (1 − *a*)exp(−*kbt*)	(36)

## Activation energy (*E_*a*_*)

*E*_*a*_ is the least amount of energy needed for drying. It is assessed from the association between sample average temperature and effective moisture diffusivity by following Arrhenius Equation (18):


(18) $$\begin{array}{l} D=\;D_{0}\exp\left(-\frac{E_{a}}{R\left(T+273.15\right)}\right) \end{array}$$

D_0_ denotes the diffusion factor (m^2^/s), R = universal gas constant (8.3145 kJ/mol). *E*_a_ = activation energy (kJ/mol), and T = sample's average temperature (135, 136). Equation (19) predicted the *E*_a_ values for various product thicknesses by drawing the fitting curve between ln D and 1/(T + 273.15).


(19) $$\begin{array}{l} Slope=\;-\;\frac{E_{a}}{R} \end{array}$$

*E*_a_ values of the SPs are tabulated in Table 6. The *Ea* results summarized in Table 6 are within the acceptable range of 12–43.26 kJ/mol for vegetables and fruits (6). Greater activation energy derives from the increased energy needed to commence moisture diffusion in a large-thick material.

**Table 6:** Activation energy and energy consumption summarized from previous studies for sweet potato drying.

**Drying conditions/Temperature (°C)**	**Size (mm)**	***R* ^2^**	**Activation energy *E_*a*_* (KJ/mol)**	**Energy consumption (MJ.kg^−1^ water)**	**References**
CHAD, IRD, IRD-CHAD; *T* = 50–70°C, 1,100–1,400 W/m^2^ IR	4–6	0.784–0.999	13.24–14.87, 12.22–18.76, 11.57–36.44	6 mm CHAD 220.39, IRD 0.34 kW h/kg 4 mm CHAD 337.79, 2.06 IRD	(34)
IR = (1,100 and 1,400 W/m^2^)	4–6	0.999	12.83–34.64	Varied from 0.91 to 4.82	(34)
HAD; *T =* 50–80°C	5, 10, 15	0.987	11.1, 30.4	–	(18)
HAD; *T =* 40–70°C; US = 0, 30, 60 W	5	–	–	Varied from 1.4 to 2.6	(47)
HAD; *T =* 50–80°C SMB, WAT, UNT treated	4	–	11.25, 9.13, 17.5	–	(49)
100 Pa MWFD, 4.5 kPa MWVD, 80°C MWSBD	10	–	–	MWFD 10,027.33, MWVD 4,259.33, MWSBD 3,004.33	(137)
HAD; *T =* 10–40°C	–	–	64.2	–	(35)
*P* = 200, 400, 600, 800 W	3.5, 5, 7, 9	–	1.621, 1.597, 1.451 and 1.423	Minimum 0.680 and maximum 2.591	(138)
HAD; *T =* 50–90°C	5, 8, 12	0.990	6.18–19.04	–	(14)
IR; *T =* 70°C	3	–	–	Minimum 81.537 and maximum 173.761	(139)
HAD; *T=* 50–90°C	3–8	–	13.48–16.50		(13)
Far-IR; *T =* 60, 70, 80°C	8, 10	–	–	8 mm was <2.42–3.41	(140)
HAD; *T =* 50–80°C	–	0.987	23.29	–	(17)
Tray, IR, FBD; *T =* 45, 55, 65°C	–	–	35.88, 33.21, and 17.33	–	(36)
HAD; *T =* 50–70°C	-	–	23.2 and 22.7	–	(64)
IR-HAD; *T =* 60–70°C	4	–	11.38	27.67–41.44	(37)
HAD; *T =* 50, 60, 70°C; IR = 1,100 W/m^2^	4	–	8.74–34.76	–	(141)
MWFD, WVD, MWSBD (−38, 4.5 KPa, 80°C)	10	–	–	–	(137)

## Specific energy consumption

When selecting a proper drying technique to minimize process costs, energy consumption during various drying methods should be considered. According to Xie et al. (139), the lowest and greater specific energy consumption were 81.537 and 173.761 (MJ kg^−1^ water) at infrared drying at 70°C, respectively. The lowest specific energy consumption (SEC) value (0.680 MJ kg^−1^ water) for drying samples was attained with a thickness of 3.5 m at a microwave power level of 200 W. Whereas, the highest value (2.591 MJ kg^−1^ water) was achieved 9 mm thick sample and a power level of 800 W (138). When drying SP, Onwude et al. (8) convective hot-air drying can consume ~337.79 (MJ kg^−1^ water) of specific energy at 70°C for 4 mm slices. According to other research findings, a lower drying temperature results in higher specific energy consumption (142). SEC was 227.39 and 265.99 (MJ kg^−1^ water) at 50 and 60°C, respectively. Infrared-assisted convective hot-air drying, on the other hand, reduced energy consumption by 69.34–85.59% (8). Limited investigations have assessed the specific energy consumption of enhanced drying approaches, such as microwave-assisted convective drying, particularly when drying SPs. Further study should be conducted using combined drying methods to determine SPs' specific energy consumption. However, SER is stated as the amount of energy consumption (EC) on the dry basis to sample mass MS (g), or the EC ratio removed from the sample during drying to water mass MW (g)


(20) $$\begin{array}{l} SER=\;\frac{E_{C}}{m_{s}} \end{array}$$

(21) $$\begin{array}{l} SER=\;\frac{E_{C}}{m_{w}} \end{array}$$

During the drying process, the SER value slowly declines as the moisture removal is significantly reduced as a result of inadequate moisture of the dried product surface.

## Major issues and future recommendation

The positive aspects of a controlled drying method include rapid drying, preservation of nutrients, color, and shelf life.

A few future research hotspots are found in detailed literature surveys mentioned below.

1. Optimization conditions could be used in dryers and quality parameters to diminish the drying time and make higher-quality foodstuffs more attractive to product developers and consumers.
2. Osmotic pretreatments, such as sugars, improve the nutritional quality while making the product difficult to dry.
3. Using US application alone or combined with osmotic pretreatment enhances the mass transfer ratio with minimal reduction in the structure and quality of SPs.
4. Combined dryers including hot air, IR, and microwave drying reduce the drying time and cost of regular dryers, i.e., cabinet or oven drying.
5. Further studies on improving quality parameters and mathematical modeling, hyperspectral imaging, FTIR, and NIR techniques might be employed as an alternative non-destructive tool for fast, accurate, and rapid determination in the drying process.
6. Further research is needed to improve model parameters or modify existing models, such as heat transfer coefficients and standardized experimental temperature measurement methods, and apply the development model to SPs.
7. Due to the increase in experimental costs, the focus is on computer-based solutions, namely online approximation of drying kinetics and drying techniques that also need to control industrial operations. To achieve more dependable and precise findings, the correctness of these simulations was determined by utilizing a suitable mathematical model for drying.

## Conclusion

This review examines sweet potato drying methods and offers the findings of previous studies. The various drying processes, drying rates, and impacts on product quality are outlined, and operational requirements for increasing drying quality are mentioned. This detailed study includes mathematical models for estimating sweet potato water ratios. A greater drying temperature, lower relative humidity, and better velocity contribute to a faster drying rate. The drying speed of sweet potatoes is also affected by their quality qualities. Osmosis dehydration minimizes the drying time, initial water content, energy consumption, and product quality and. chemical pretreatments can improve the drying procedure and uphold food quality. Furthermore, novel non-thermal methods, such as ultrasonic waves, have been developed as alternative pretreatments for reducing drying periods and improving quality.

Natural drying procedures, such as sun, shadow, and wind drying, are low-cost and ecologically benign. Electric heating dryers (oven dryers) consume a lot of energy and only have a limited impact on product quality. Infrared and microwave dryers are resources and provide total control over the quality of dried items. The relative humidity and drying temperatures can be modified to get the final product with minimal nutritional loss. Low relative humidity, medium air temperatures (40–70°C), and high velocity are critical operating parameters for an effective drying operation. Several scholars have presented mathematical models of sweet potato drying, including the Page, Hii, and Midilli models, which give the optimum fitting when characterizing sweet potato drying behavior. Most recent research has found that drying sweet potatoes at lower temperatures preserves their nutrients.

The future challenge in food drying is to optimize the dryers and reduce both the equipment cost and running cost to improve the quality of the product and to use renewable energy for drying to reduce emissions. As the expense of trials rises, more emphasis is being placed on computer-based solutions such as FTIR online assessment of quality parameters, NIR spectroscopy to simulate drying difficulties, and selecting the appropriate dryer.

## Author contributions

All authors listed have made a substantial, direct, and intellectual contribution to the work and approved it for publication.

## Funding

This study was supported by the National Natural Science Foundation of China (U1904110 and 32172259), the Postdoctoral Research Project from Henan Province (2021), the Science and Technology Project of Henan Province (212300410033), the Program for the Top Young Talents of Henan Associate for Science and Technology (2021), and the Innovative Funds Plan of Henan University of Technology (2021ZKCJ03).

## Conflict of interest

The authors declare that the research was conducted in the absence of any commercial or financial relationships that could be construed as a potential conflict of interest.

## Publisher's note

All claims expressed in this article are solely those of the authors and do not necessarily represent those of their affiliated organizations, or those of the publisher, the editors and the reviewers. Any product that may be evaluated in this article, or claim that may be made by its manufacturer, is not guaranteed or endorsed by the publisher.

## References

[1] Tayyab Rashid M Ahmed Jatoi M Safdar B Wali A Muhammad Aadil R Sarpong F Ma H. Modeling the drying of ultrasound and glucose pretreated sweet potatoes: the impact on phytochemical and functional groups. Ultrason Sonochem. (2020) 68:e105226. 10.1016/j.ultsonch.2020.105226 32599166

[2] Santos PHS Silva MA. Retention of vitamin C in drying processes of fruits and vegetables—a review. Dry Technol. (2008) 26:1421–37. 10.1080/07373930802458911

[3] Tang Y Cai W Xu B. Profiles of phenolics, carotenoids and antioxidative capacities of thermal processed white, yellow, orange and purple sweet potatoes grown in Guilin, China. Food Sci Hum Wellness. (2015) 4:123–32. 10.1016/j.fshw.2015.07.003

[4] Sagar VR Suresh Kumar P. Recent advances in drying and dehydration of fruits and vegetables: a review. J Food Sci Technol. (2010) 47:15–26. 10.1007/s13197-010-0010-8 23572596 PMC3550996

[5] Mohd Hanim AB Chin NL Yusof YA. Physico-chemical and flowability characteristics of a new variety of Malaysian sweet potato, VitAto flour. Int Food Res J. (2014) 21:2099–107.

[6] Onwude DI Hashim N Janius RB Nawi NM Abdan K. Modeling the thin-layer drying of fruits and vegetables: a review. Compr Rev Food Sci Food Saf. (2016) 15:599–618. 10.1111/1541-4337.12196 33401820

[7] Akpinar EK. Mathematical modelling of thin layer drying process under open sun of some aromatic plants. J Food Eng. (2006) 77:864–70. 10.1016/j.jfoodeng.2005.08.014

[8] Onwude DI Hashim N Chen G. Recent advances of novel thermal combined hot air drying of agricultural crops. Trends Food Sci Technol. (2016) 57:132–45. 10.1016/j.tifs.2016.09.012

[9] Wang Y Li X Chen X Li B Mao X Miao J. Effects of hot air and microwave-assisted drying on drying kinetics, physicochemical properties, and energy consumption of chrysanthemum. Chem Eng Process Process Intens. (2018) 129:84–94. 10.1016/j.cep.2018.03.020

[10] Raghavi LM Moses JA Anandharamakrishnan C. Refractance window drying of foods: a review. J Food Eng. (2018) 222:267–75. 10.1016/j.jfoodeng.2017.11.03230207393

[11] Zhang M Tang J Mujumdar AS Wang S. Trends in microwave-related drying of fruits and vegetables. Trends Food Sci Technol. (2006) 17:524–34. 10.1016/j.tifs.2006.04.011

[12] Demirhan E Özbek B. Thin-layer drying characteristics and modeling of celery leaves undergoing microwave treatment. Chem Eng Commun. (2011) 198:957–75. 10.1080/00986445.2011.545298

[13] Fan K Chen L He J Yan F. Characterization of thin layer hot air drying of sweet potatoes (*I pomoea batatas* L.) slices. J Food Process Preserv. (2015) 39:1361–71. 10.1111/jfpp.12355

[14] Singh N Pandey R. Convective air drying characteristics of sweet potato (*Ipomoea batatas* L.). Food Bioprod Process. (2012) 90:317–22. 10.1016/j.fbp.2011.06.006

[15] Hatamipour MS Kazemi HH Nooralivand A Nozarpoor A. Drying characteristics of six varieties of sweet potatoes in different dryers. Food Bioprod Process. (2007) 85:171–7. 10.1205/fpb07032

[16] Bechoff A Tomlins KI Dhuique-Mayer C Dufour D Westby A. Understanding carotenoid losses in orange-fleshed sweet potato in drying and storage. In: 15th Trienn Symp Int Soc Trop Root Crop (ISTRC) Trop Roots Tubers a Chang Clim A convement Oppor World. (2009).

[17] Zhu A Jiang F. Modeling of mass transfer performance of hot-air drying of sweet potato (*Ipomoea batatas* L) slices. Chem Ind Chem Eng Q. (2014) 20:171–81. 10.2298/CICEQ120509122Z

[18] Falade KO Solademi OJ. Modelling of air drying of fresh and blanched sweet potato slices. Int J Food Sci Technol. (2010) 45:278–88. 10.1111/j.1365-2621.2009.02133.x

[19] Sun Y Liu Y Yu H Xie A Li X Yin Y. Non-destructive prediction of moisture content and freezable water content of purple-fleshed sweet potato slices during drying process using hyperspectral imaging technique. Food Anal Methods. (2017) 10:1535–46. 10.1007/s12161-016-0722-0

[20] Singh NJ Pandey RK. Neural network approaches for prediction of drying kinetics during drying of sweet potato. Agric Eng Int CIGR J. (2011) 13.

[21] Soison B Jangchud K Jangchud A Harnsilawat T Piyachomkwan K Charunuch C. Physico-functional and antioxidant properties of purple-flesh sweet potato flours as affected by extrusion and drum-drying treatments. Int J Food Sci Technol. (2014) 49:2067–75. 10.1111/ijfs.12515

[22] Doymaz I. Experimental study and mathematical modeling of thin-layer infrared drying of watermelon seeds. J Food Process Preserv. (2014) 38:1377–84. 10.1111/jfpp.12217

[23] Olawale AS Omole SO. Thin layer drying models for sweet potato in tray dryer. Agric Eng Int CIGR J. (2012) 14:131–137.

[24] Jeng TL Lai CC Liao TC Lin SY Sung JM. Effects of drying on caffeoylquinic acid derivative content and antioxidant capacity of sweet potato leaves. J Food Drug Anal. (2015) 23:701–8. 10.1016/j.jfda.2014.07.002 28911486 PMC9345467

[25] Miranda M Maureira H Rodríguez K Vega-Gálvez A. Influence of temperature on the drying kinetics, physicochemical properties, and antioxidant capacity of Aloe Vera (*Aloe barbadensis* Miller) gel. J Food Eng. (2009) 91:297–304. 10.1016/j.jfoodeng.2008.09.007

[26] Peng Z Li J Guan Y Zhao G. Effect of carriers on physicochemical properties, antioxidant activities and biological components of spray-dried purple sweet potato flours. LWT Food Sci Technol. (2013) 51:348–55. 10.1016/j.lwt.2012.09.022

[27] Nicanuru C Laswai HS Sila DN. Effect of pre-treatments and drying on nutrient content of orange fleshed sweet potatoes in Maswa District, Tanzania. In: Fifth African High Educ Week RUFORUM Bienn Conf 2016, “Linking Agric Univ with Civ Soc Priv Sect Gov other stakeholders Support Agric Dev Africa.” Cape Town, South Afr. (2016), p. 923–9. Available online at: http://ir.jkuat.ac.ke/handle/123456789/2049 (accessed December 31, 2021).

[28] Bengtsson A Namutebi A Alminger ML Svanberg U. Effects of various traditional processing methods on the all-trans-β-carotene content of orange-fleshed sweet potato. J Food Compos Anal. (2008) 21:134–43. 10.1016/j.jfca.2007.09.006

[29] Oh S Ramachandraiah K Hong GP. Effects of pulsed infra-red radiation followed by hot-press drying on the properties of mashed sweet potato chips. LWT Food Sci Technol. (2017) 82:66–71. 10.1016/j.lwt.2017.04.023

[30] Ahmed M Akter MS Lee JC Eun JB. Encapsulation by spray drying of bioactive components, physicochemical and morphological properties from purple sweet potato. LWT Food Sci Technol. (2010) 43:1307–12. 10.1016/j.lwt.2010.05.014

[31] Onwude DI Hashim N Abdan K Janius R Chen G. Modelling the mid-infrared drying of sweet potato: kinetics, mass and heat transfer parameters, and energy consumption. Heat Mass Transf Stoffuebertragung. (2018) 54:2917–33. 10.1007/s00231-018-2338-y

[32] Yan WQ Zhang M Huang LL Mujumdar AS Tang J. Influence of microwave drying method on the characteristics of the sweet potato dices. J Food Process Preserv. (2013) 37:662–9. 10.1111/j.1745-4549.2012.00707.x

[33] Doymaz I. Infrared drying of sweet potato (*Ipomoea batatas* L.) slices. J Food Sci Technol. (2012) 49:760–6. 10.1007/s13197-010-0217-8 24293696 PMC3550829

[34] Onwude DI Hashim N Abdan K Janius R Chen G Kumar C. Modelling of coupled heat and mass transfer for combined infrared and hot-air drying of sweet potato. J Food Eng. (2018) 228:12–24. 10.1016/j.jfoodeng.2018.02.006

[35] Bechoff A. Investigating Carotenoid Loss After Drying and Storage of Orange-Fleshed Sweet Potato. London: University of Greenich (2010).

[36] Thao HM Noomhorm A. Modeling and effects of various drying methods on sweet potato starch properties. Walailak J. (2011) 8:139–58.

[37] Onwude DI Hashim N Abdan K Janius R Chen G. Investigating the influence of novel drying methods on sweet potato (*Ipomoea batatas* L.): kinetics, energy consumption, color, and microstructure. J Food Process Eng. (2018) 41:e12686. 10.1111/jfpe.12686

[38] Onwude DI Hashim N Abdan K Janius R Chen G. Numerical modeling of radiative heat and mass transfer for sweet potato during drying. J Food Process Preserv. (2018) 42:1–14. 10.1111/jfpp.13741

[39] Bechoff A Westby A Menya G Tomlins KI. Effect of pretreatments for retaining total carotenoids in dried and stored orange-fleshed-sweet potato chips. J Food Qual. (2011) 34:259–67. 10.1111/j.1745-4557.2011.00391.x

[40] Mwanri A Kogi-Makau W Laswai H. Nutrients and antinutrients composition of raw, cooked and sun-dried sweet potato leaves. Afric J Food Agric Nutr Dev. (2011) 11:5142–56. 10.4314/ajfand.v11i5.70442

[41] Liu P Zhang M Mujumdar AS. Purple-fleshed sweet potato cubes drying in a microwave-assisted spouted bed dryer. Dry Technol. (2014) 32:1865–71. 10.1080/07373937.2014.953174

[42] Xu S Pegg RB Kerr WL. Sensory and physicochemical properties of sweet potato chips made by vacuum-belt drying. J Food Process Eng. (2013) 36:353–63. 10.1111/jfpe.12002

[43] Onwude DI Hashim N Abdan K Janius R Chen G. Experimental studies and mathematical simulation of intermittent infrared and convective drying of sweet potato (*Ipomoea batatas* L). Food Bioprod Process. (2019) 114:163–74. 10.1016/j.fbp.2018.12.006

[44] Rashid MT Ma H Jatoi MA Safdar B El-Mesery HS Sarpong F Ali Z Wali A. Multi-frequency ultrasound and sequential infrared drying on drying kinetics, thermodynamic properties, and quality assessment of sweet potatoes. J Food Process Eng. (2019) 42:13127. 10.1111/jfpe.13127

[45] Zhao D Wang Y Zhu Y Ni Y. Effect of carbonic maceration pre-treatment on the drying behavior and physicochemical compositions of sweet potato dried with intermittent or continuous microwave. Dry Technol. (2016) 34:1604–12. 10.1080/07373937.2016.1138231

[46] Abe S Takimoto S Yamamuro Y Tau K Takenaga F Suzuki K. High-pressure and heat pretreatment effects on rehydration and quality of sweet potato. Am J Food Technol. (2011) 6:63–71. 10.3923/ajft.2011.63.71

[47] DLiu Y Sun Y Yu H Yin Y Li H Duan X. Hot air drying of purple-fleshed sweet potato with contact ultrasound assistance. Dry Technol. (2016) 35:564–76. 10.1080/07373937.2016.1193867

[48] Liu Y Sun Y Xie A Yu H Yin Y Li X. Potential of hyperspectral imaging for rapid prediction of anthocyanin content of purple-fleshed sweet potato slices during drying process. Food Anal Methods. (2017) 10:3836–46. 10.1007/s12161-017-0950-y

[49] Dinrifo RR. Effects of pre-treatments on drying kinetics of sweet potato slices. Agric Eng Int CIGR J. (2012) 14:136–45.

[50] Ahmed M Sorifa AM Eun JB. Effect of pretreatments and drying temperatures on sweet potato flour. Int J Food Sci Technol. (2010) 45:726–32. 10.1111/j.1365-2621.2010.02191.x30258617

[51] Ahmed M Akter MS Eun JB. Optimisation of drying conditions for the extraction of β-carotene, phenolic and ascorbic acid content from yellow-fleshed sweet potato using response surface methodology. Int J Food Sci Technol. (2011) 46:1356–62. 10.1111/j.1365-2621.2011.02612.x 20858156

[52] Bechoff A Tomlins K Dhuique-Mayer C Dove R Westby A. On-farm evaluation of the impact of drying and storage on the carotenoid content of orange-fleshed sweet potato (*Ipomea batata* Lam). Int J Food Sci Technol. (2011) 46:52–60. 10.1111/j.1365-2621.2010.02450.x

[53] Md. Nahid Hossain Jany Md. Anisur Rahman Mazumder MBU. Effect of varietal differences on the osmotic dehydration of sweet potatoes (*Ipomoea batatas* Lam). Int J Agric Food Sci. (2016) 6:4–18.

[54] Clifford IO Kingsley E Chika CO Chinyere II. Effects of osmotic dewatering and oven drying on β-carotene content of sliced light yellow-fleshed sweet potato (*Ipomea batatas* L). Niger Food J. (2014) 32:25–32. 10.1016/S0189-7241(15)30114-4

[55] Antonio GC Azoubel PM Murr FEX Park KJ. Osmotic dehydration of sweet potato (*Ipomoea batatas*) in ternary solutions. Ciência e Tecnol Aliment. (2008) 28:696–701. 10.1590/s0101-20612008000300028

[56] Antonio GC Alves DG Azoubel PM Murr FEX Park KJ. Influence of osmotic dehydration and high temperature short time processes on dried sweet potato (*Ipomoea batatas* Lam). J Food Eng. (2008) 84:375–82. 10.1016/j.jfoodeng.2007.05.033

[57] Lagnika C Huang J Jiang N Li D Liu C Song J Wei Q Zhang M. Ultrasound-assisted osmotic process on quality of microwave vacuum drying sweet potato. Dry Technol. (2018) 36:1367–1379. 10.1080/07373937.2017.1402786

[58] Junqueira JRJ Mendonça KS Corrêa JLG. Microwave drying of sweet potato (*Ipomoea batatas* L.) slices: influence of the osmotic pretreatment defect. Diffus Forum. (2016) 367:167–74. 10.4028/www.scientific.net/ddf.367.167

[59] Duc LA Han JW Keum DH. Thin layer drying characteristics of rapeseed (*Brassica napus* L.). J Stored Prod Res. (2011) 47:32–8. 10.1016/j.jspr.2010.05.006

[60] Ogawa T Chuma A Aimoto U Adachi S. Effects of drying temperature and relative humidity on spaghetti characteristics. Dry Technol. (2017) 35:1214–24. 10.1080/07373937.2016.1236812

[61] Sabudin S Hakimi Remlee MZ Mohideen Batcha MF. Effect of relative humidity on drying kinetics of agricultural products. Appl Mech Mater. (2014) 699:257–62. 10.4028/www.scientific.net/amm.699.257

[62] Walker JCF. Primary Wood Processing: Principles and Practice. Berlin: Springer (2006). p. 1–596. 10.1007/1-4020-4393-7

[63] Singh S Raina CS Bawa AS Saxena DC. Effect of pretreatments on drying and rehydration kinetics and color of sweet potato slices. Dry Technol. (2006) 24:1487–94. 10.1080/07373930600952834

[64] Doymaz I. Thin-layer drying characteristics of sweet potato slices and mathematical modelling. Heat Mass Transf Stoffuebertragung. (2011) 47:277–85. 10.1007/s00231-010-0722-3

[65] Mujumdar AS. Handbook of Industrial Drying. Milton Park: Taylor and Francis (2006).

[66] Abbasi Souraki B Mowla D. Simulation of drying behaviour of a small spherical foodstuff in a microwave assisted fluidized bed of inert particles. Food Res Int. (2008) 41:255–65. 10.1016/j.foodres.2007.12.008

[67] Hatamipour MS Mowla D. Experimental and theoretical investigation of drying of carrots in a fluidized bed with energy carrier. Dry Technol. (2003) 21:83–101. 10.1081/DRT-120017285

[68] Zhang M Chen D. Effects of low temperature soaking on color and texture of green eggplants. J Food Eng. (2006) 74:54–9. 10.1016/j.jfoodeng.2005.02.015

[69] Beigi M. Energy efficiency and moisture diffusivity of apple slices during convective drying. Food Sci Technol. (2016) 36:145–50. 10.1590/1678-457X.0068

[70] Kosambo L. Effect of storage and processing on all trans-β carotene content in fresh Sweet potato (*Ipomoea batatas* Lam) roots and its products. In: CIP Funded Research Project: Annual Report (July 2003–June 2004). Nairobi: Kenya Industrial Research and Development Institute (2004). p. 11.

[71] Lohachoompol V Srzednicki G Craske J. The change of total anthocyanins in blueberries and their antioxidant effect after drying and freezing. J Biomed Biotechnol. (2004) 2004:248–52. 10.1155/S1110724304406123 15577185 PMC1082901

[72] Joykumar Singh N Pandey RK. Convective air drying characteristics of sweet potato cube (*Ipomoea batatas* L). Food Bioprod Process. (2012) 90:317–22. 10.1016/j.fbp.2011.06.006

[73] Potisate Y Phoungchandang S Kerr WL. The effects of predrying treatments and different drying methods on phytochemical compound retention and drying characteristics of Moringa leaves (*Moringa oleifera* Lam). Dry Technol. (2014) 32:1970–85. 10.1080/07373937.2014.926912

[74] Li Z Raghavan GSV Orsat V. Optimal power control strategies in microwave drying. J Food Eng. (2010) 99:263–8. 10.1016/j.jfoodeng.2010.02.024

[75] Huang LL Zhang M Mujumdar AS Lim RX. Comparison of four drying methods for re-structured mixed potato with apple chips. J Food Eng. (2011) 103:279–84. 10.1016/j.jfoodeng.2010.10.025

[76] Giri SK Prasad S. Drying kinetics and rehydration characteristics of microwave-vacuum and convective hot-air dried mushrooms. J Food Eng. (2007) 78:512–21. 10.1016/j.jfoodeng.2005.10.021

[77] Marzuki SU Pranoto Y Khumsap T Nguyen LT. Effect of blanching pretreatment and microwave-vacuum drying on drying kinetics and physicochemical properties of purple-fleshed sweet potato. J Food Sci Technol. (2021) 58:2884–95. 10.1007/s13197-020-04789-5 34294950 PMC8249554

[78] Lagnika C Jiang N Song J Li D Liu C Wei Q. Effects of pretreatments on properties of microwave-vacuum drying of sweet potato slices. Dry Technol. (2019) 0:1–14. 10.1080/07373937.2018.1543702

[79] Monteiro RL de Moraes JO Domingos JD Carciofi BAM Laurindo JB. Evolution of the physicochemical properties of oil-free sweet potato chips during microwave vacuum drying. Innov Food Sci Emerg Technol. (2020) 63:102317. 10.1016/j.ifset.2020.102317

[80] Gabel MM Pan Z Amaratunga KSP Harris LJ Thompson JF. Catalytic infrared dehydration of onions. J Food Sci. (2006) 71:E351–7. 10.1111/j.1750-3841.2006.00170.x34677930

[81] ffah-Manu L Oduro I Addo A. Effect of dextrinized sweet potatoes on the physicochemical and sensory quality of infra-red dried mango leather. J Food Process Technol. (2013) 4:5. 10.4172/2157-7110.1000230

[82] Okilya S Mukisa IM Kaaya AN. Effect of solar drying on the quality and acceptability of jackfruit leather. Electron J Environ Agric Food Chem. (2010) 9:101–11.

[83] Rashid MT Ma H Jatoi MA Wali A El-Mesery HS Ali Z. Effect of infrared drying with multifrequency ultrasound pretreatments on the stability of phytochemical properties, antioxidant potential, and textural quality of dried sweet potatoes. J Food Biochem. (2019) 43:e12809. 10.1111/jfbc.12809 31353587

[84] Coskun C Oktay Z Dincer I. Modified exergoeconomic modeling of geothermal power plants. Energy. (2011) 36:6358–66. 10.1016/j.energy.2011.09.038

[85] Yang J Chen JF Zhao YY Mao LC. Effects of drying processes on the antioxidant properties in sweet potatoes. Agric Sci China. (2010) 9:1522–9. 10.1016/S1671-2927(09)60246-7

[86] Sun J Li R Zhu H. Water changes of purple sweet potato slices using low-field nmr during vacuum freeze drying. (2021) 42:9–14.

[87] Deng ZJ Jiang HT. Different drying methods on quality of the purple sweet potato flour. Sci Technol food Ind. (2011) 12:359–64.

[88] Hiranvarachat B Devahastin S Chiewchan N. Effects of acid pretreatments on some physicochemical properties of carrot undergoing hot air drying. Food Bioprod Process. (2011) 89:116–27. 10.1016/j.fbp.2010.03.010

[89] de Junqueira JR Corréa JLG de Mendonça KS. Evaluation of the shrinkage effect on the modeling kinetics of osmotic dehydration of sweet potato (*Ipomoea batatas* L.). J Food Process Preserv. (2017) 41:e12881. 10.1111/jfpp.12881

[90] Kuyu CG Tola YB Mohammed A Ramaswamy HS. Determination of citric acid pretreatment effect on nutrient content, bioactive components, and total antioxidant capacity of dried sweet potato flour. Food Sci Nutr. (2018) 6:1724–33. 10.1002/fsn3.747 30258617 PMC6145308

[91] Ruttarattanamongkol K Chittrakorn S Weerawatanakorn M Dangpium N. Effect of drying conditions on properties, pigments and antioxidant activity retentions of pretreated orange and purple-fleshed sweet potato flours. J Food Sci Technol. (2016) 53:1811–22. 10.1007/s13197-015-2086-7 27413208 PMC4926894

[92] Singh S Raina CS Bawa AS Saxena DC. Optimisation of processing variables in the preparation of sweet potato chips using response surface methodology. Eur Food Res Technol. (2003) 217:374–81. 10.1007/s00217-003-0768-2

[93] Tesniere C Flanzy C. Carbonic maceration wines: characteristics and winemaking process. Adv Food Nutr Res. (2011) 63:1–15. 10.1016/B978-0-12-384927-4.00001-4 21867890

[94] Liu L Wang Y Zhao D An K Ding S Wang Z. Effect of carbonic maceration pre-treatment on drying kinetics of chilli (*Capsicum annuum* L.) flesh and quality of dried product. Food Bioprocess Technol. (2014) 7:2516–27. 10.1007/s11947-014-1253-6

[95] Wang Y Tao H Yang J An K Ding S Zhao D Wang Z. Effect of carbonic maceration on infrared drying kinetics and raisin qualities of Red Globe (*Vitis vinifera* L.): a new pre-treatment technology before drying. Innov Food Sci Emerg Technol. (2014) 26:462–8. 10.1016/j.ifset.2014.09.001

[96] Rahman MS. Food Preservation and Processing Methods. 1st edn. In: Kalyani, editor. (Elseiver: New Delhi) (2012). p. 1–17.

[97] Ciurzyńska A Kowalska H Czajkowska K Lenart A. Osmotic dehydration in production of sustainable and healthy food. Trends Food Sci Technol. (2016) 50:186–92. 10.1016/j.tifs.2016.01.017

[98] Biswal RN Wilhelm LR Rojas A Mount JR. Moisture diffusivity in osmotically concentrated diced sweet potato during air drying. Trans ASAE. (1997) 40:1383–90.

[99] Genina-Soto P Barrera-Cortes J Gutierrez-Lopez G Nieto EA. Temperature and concentration effects of osmotic media on OD profiles of sweet potato cubes. Dry Technol. (2001) 19:547–58. 10.1081/DRT-100103933

[100] Rashid MT Ma H Safdar B Jatoi MA Wali A Sarpong F Zhou C. Synergy of ultrasound and osmotic dehydration in improving drying kinetics and quality of dried sweet potato (*Ipomea batatas* L.). J Food Saf Food Qual. (2019) 70:72–81.

[101] Rashid MT Ma H Jatoi MA Hashim MM Wali A Safdar B. Influence of ultrasonic pretreatment with hot air drying on nutritional quality and structural related changes in dried sweet potatoes. Int J Food Eng. (2019) 15. 10.1515/ijfe-2018-0409

[102] Lagnika C Riaz A Jiang N Song J Li D Liu C Wei Q Zhang M. Effects of pretreatment and drying methods on the quality and stability of dried sweet potato slices during storage. J Food Process Preserv. (2021) 45:15807. 10.1111/JFPP.15807

[103] Infante RA Azoubel PM de Lima MAB Stamford TCM Araújo AS de Mendonça WS. Ultrasound pretreatment application in dehydration: its influence on the microstructure, antioxidant activity and carotenoid retention of biofortified Beauregard sweet potato (*Ipomoea batatas* Lam). J Food Sci Technol. (2021) 58:4542–9. 10.1007/S13197-020-04938-W 34629518 PMC8479030

[104] Corrêa JLG Pereira LM Vieira GS Hubinger MD. Mass transfer kinetics of pulsed vacuum osmotic dehydration of guavas. J Food Eng. (2010) 96:498–504. 10.1016/j.jfoodeng.2009.08.032

[105] Ruiz-López II Ruiz-Espinosa H Herman-Lara E Zárate-Castillo G. Modeling of kinetics, equilibrium and distribution data of osmotically dehydrated carambola (*Averrhoa carambola* L) in sugar solutions. J Food Eng. (2011) 104:218–26. 10.1016/j.jfoodeng.2010.12.013

[106] Brochier B Marczak LDF Noreña CPZ. Use of different kinds of solutes alternative to sucrose in osmotic dehydration of yacon. Brazilian Arch Biol Technol. (2015) 58:34–40. 10.1590/S1516-8913201400035

[107] de Mendonça KS Corrêa JLG de Jesus Junqueira JR Pereira MC de A Vilela MB. Optimization of osmotic dehydration of yacon slices. Dry Technol. (2016) 34:386–94. 10.1080/07373937.2015.1054511

[108] Zhang Z Wheatley CC Corke H. Biochemical changes during storage of sweet potato roots differing in dry matter content. Postharvest Biol Technol. (2002) 24:317–25. 10.1016/S0925-5214(01)00149-1

[109] Wang Y-Y Yan J-K Rashid MT Ding Y Chikari F Huang S Ma H. Dual-frequency sequential ultrasound thawing for improving the quality of quick-frozen small yellow croaker and its possible mechanisms. Innov Food Sci Emerg Technol. (2021) 68:102614. 10.1016/j.ifset.2021.102614

[110] Lv JM Gouda M Zhu YY Ye XQ Chen JC. Ultrasound-assisted extraction optimization of proanthocyanidins from kiwi (Actinidia chinensis) leaves and evaluation of its antioxidant activity. Antioxidants. (2021) 10:184–91. 10.3390/antiox10081317 34439565 PMC8389255

[111] Gouda M El-Din Bekhit A Tang Y Huang Y Huang L He Y Li X. Recent innovations of ultrasound green technology in herbal phytochemistry: a review. Ultrason Sonochem. (2021) 73:105538. 10.1016/j.ultsonch.2021.105538 33819867 PMC8048006

[112] Chen Y Sheng L Gouda M Ma M. Studies on foaming and physicochemical properties of egg white during cold storage. Colloids Surfaces A Physicochem Eng Asp. (2019) 582:123916. 10.1016/j.colsurfa.2019.123916

[113] Haile F Admassu S Fisseha A. Effects of pre-treatments and drying methods on chemical composition, microbial and sensory qualities of orange-fleshed sweet potato flour and porridge. Am J Food Sci Technol. (2015) 3:82–8. 10.12691/ajfst-3-3-5

[114] Nicanuru C Laswai HS Sila DN. Effect of sun-drying on nutrient content of orange fleshed sweet potato tubers in Tanzania. Sky J Food Sci. (2015) 4:91–101.

[115] Ramesh Yadav A Guha M Tharanathan RN Ramteke RS. Changes in characteristics of sweet potato flour prepared by different drying techniques. LWT Food Sci Technol. (2006) 39:20–6. 10.1016/j.lwt.2004.12.010

[116] Woolfe JA. Sweet Potato: An Untapped Food Resource. (1992). Cambridge: Cambridge University Press.

[117] Motevali A Minaei S Khoshtaghaza MH Amirnejat H. Comparison of energy consumption and specific energy requirements of different methods for drying mushroom slices. Energy. (2011) 36:6433–41. 10.1016/j.energy.2011.09.024

[118] Olatunde GO Henshaw FO Idowu MA Tomlins K. Quality attributes of sweet potato flour as influenced by variety, pretreatment and drying method. Food Sci Nutr. (2016) 4:623–35. 10.1002/fsn3.325 27386111 PMC4930505

[119] El-Beltagy A Gamea GR Essa AHA. Solar drying characteristics of strawberry. J Food Eng. (2007) 78:456–64. 10.1016/j.jfoodeng.2005.10.015

[120] Akoy EOM. Experimental characterization and modeling of thin-layer drying of mango slices. Int Food Res J. (2014) 21:1911–7.

[121] Hashim N Daniel O Rahaman E. A preliminary study: kinetic model of drying process of pumpkins (*Cucurbita moschata*) in a convective hot air dryer. Agric Agric Sci Procedia. (2014) 2:345–52. 10.1016/j.aaspro.2014.11.048

[122] Zenoozian MS Feng H Razavi SMA Shahidi F Pourreza HR. Image analysis and dynamic modeling of thin-layer drying of osmotically dehydrated pumpkin. J Food Process Preserv. (2008) 32:88–102. 10.1111/j.1745-4549.2007.00167.x

[123] Kulwinder KAK. Drying kinetics and quality characteristics of beetroot slices under hot air followed by microwave finish drying. African J Agric Res. (2014) 9:1036–44. 10.5897/AJAR2013

[124] Campus M Bellevue A. Mathematical modeling of thin layer drying kinetics of apples slices. Int Food Res J. (2006) 19:1949–58. 10.1051/IUFoST

[125] Sacilik K. Effect of drying methods on thin-layer drying characteristics of hull-less seed pumpkin (*Cucurbita pepo* L.). J Food Eng. (2007) 79:23–30. 10.1016/j.jfoodeng.2006.01.023

[126] Hii CL Law CL Cloke M. Modeling using a new thin layer drying model and product quality of cocoa. J Food Eng. (2009) 90:191–8. 10.1016/j.jfoodeng.2008.06.022

[127] Stoilova I Krastanov A Stoyanova A Denev P Gargova S Pérez-Rosés R. Comparison of different drying methods on Chinese ginger (*Zingiber officinale* Roscoe): changes in volatiles, chemical profile, antioxidant properties, and microstructure. Food Chem. (2016) 77:4716–24. 10.1016/j.phytochem.2015.07.012 26675871

[128] Gan PL Poh PE. Investigation on the effect of shapes on the drying kinetics and sensory evaluation study of dried jackfruit. Int J Sci Eng. (2014) 7:193–8. 10.12777/ijse.7.2.193-198

[129] Doymaz I Karasu S Baslar M. Effects of infrared heating on drying kinetics, antioxidant activity, phenolic content, and color of jujube fruit. J Food Meas Charact. (2016) 10:283–91. 10.1007/s11694-016-9305-4

[130] Omolola AO Jideani AIO Kapila PF. Modeling microwave drying kinetics and moisture diffusivity of mabonde banana variety. Int J Agric Biol Eng. (2014) 7:107–13. 10.3965/j.ijabe.20140706.013

[131] da Silva WP e Silva CMDPS Gama FJA Gomes JP. Mathematical models to describe thin-layer drying and to determine drying rate of whole bananas. J Saudi Soc Agric Sci. (2014) 13:67–74. 10.1016/j.jssas.2013.01.003

[132] Jena S Das H. Modelling for vacuum drying characteristics of coconut presscake. J Food Eng. (2007) 79:92–9. 10.1016/j.jfoodeng.2006.01.032

[133] Sharma GP Prasad S. Effective moisture diffusivity of garlic cloves undergoing microwave-convective drying. J Food Eng. (2004) 65:609–17. 10.1016/j.jfoodeng.2004.02.027

[134] Azizpour M Mohebbi M Hossein Haddad Khodaparast M Varidi M. Optimization of foaming parameters and investigating the effects of drying temperature on the foam-mat drying of shrimp (*Penaeus indicus*). Dry Technol. (2014) 32:374–84. 10.1080/07373937.2013.794829

[135] Chen NN Chen MQ Fu BA Song JJ. Far-infrared irradiation drying behavior of typical biomass briquettes. Energy. (2017) 121:726–38. 10.1016/j.energy.2017.01.054

[136] Hebbar UH Ramesh MN. Optimisation of processing conditions for infrared drying of cashew kernels with testa. J Sci Food Agric. (2005) 85:865–71. 10.1002/jsfa.2045

[137] Liu P Zhang M Mujumdar AS. Comparison of three microwave-assisted drying methods on the physiochemical, nutritional and sensory qualities of re-structured purple-fleshed sweet potato granules. Int J Food Sci Technol. (2012) 47:141–7. 10.1111/j.1365-2621.2011.02819.x

[138] Azimi-Nejadian H Hoseini SS. Study the effect of microwave power and slices thickness on drying characteristics of potato. Heat Mass Transf und Stoffuebertragung. (2019) 55:2921–30. 10.1007/s00231-019-02633-x

[139] Xie SY Yang ZR Yang L Li ST Wang JL Yang MJ. Performance analysis and technology optimization of infrared drying of sweet potato slice. INMATEH - Agric Eng. (2021) 64:119–30. 10.35633/inmateh-64-11

[140] Lee D Lee JH Shin YS Cho BH Han CS. Far infrared drying characteristics of the microwave-steamed sweet potato. J Biosyst Eng. (2019) 44:187–93. 10.1007/s42853-019-00028-8

[141] Onwude DI Hashim N Abdan K Janius R Chen G. The effectiveness of combined infrared and hot-air drying strategies for sweet potato. J Food Eng. (2019) 241:75–87. 10.1016/j.jfoodeng.2018.08.008

[142] Tekin ZH Başlar M Karasu S Kilicli M. Dehydration of green beans using ultrasound-assisted vacuum drying as a novel technique: drying kinetics and quality parameters. J Food Process Preserv. (2017) 41:e13227. 10.1111/jfpp.13227

